# Alanine catabolism as a targetable vulnerability for MYC-driven liver cancer

**DOI:** 10.1016/j.celrep.2026.117107

**Published:** 2026-03-17

**Authors:** Tonatiuh Montoya, Joyce V. Lee, Longhui Qiu, Abigail Krall, Nedas Matulionis, Yurim Seo, Brian N. Finck, Robin K. Kelley, Heather Christofk, Andrei Goga

**Affiliations:** 1 Department of Cell & Tissue Biology, University of California, San Francisco, San Francisco, CA, USA; 2 Biomedical Sciences Graduate Program, University of California, San Francisco, San Francisco, CA, USA; 3 Department of Medicine, University of California, San Francisco, San Francisco, CA, USA; 4 Department of Biological Chemistry, University of California, Los Angeles, Los Angeles, CA, USA; 5 Department of Medicine, Washington University, St. Louis, MO, USA; 6 UCSF Helen Diller Comprehensive Cancer Center, San Francisco, CA, USA; 7 Division of Hematology-Oncology, Department of Medicine, University of California, San Francisco, San Francisco, CA, USA; 8 Lead contact

## Abstract

Liver cancer is a leading cause of cancer-related death due to the shortage of effective therapies, and MYC overexpression defines an aggressive and difficult-to-treat subset of patients. Given MYC’s ability to reprogram cancer metabolism and the liver’s role in coordinating systemic metabolism, we hypothesized that MYC induces metabolic dependencies that could be targeted to attenuate tumor growth. We discovered that MYC-driven liver cancers catabolize alanine in a GPT2-dependent manner. GPT2 is the predominant alanine-catabolizing enzyme expressed in MYC-driven liver tumors and genetic ablation of GPT2 limited liver tumorigenesis. *In vivo* isotope tracing identified alanine as a substrate for a repertoire of pathways including the tricarboxylic acid cycle and biosynthesis. Finally, treating a MYC-driven liver tumor model with L-cycloserine diminished the frequency of mouse tumor formation and attenuated the growth of established human liver tumors. Thus, we identify a targetable metabolic dependency that MYC-driven liver tumors usurp to ensure their survival.

## In brief

Montoya et al. uncover a role for alanine as a conserved metabolic substrate for MYC-driven liver cancer. GPT2 is a therapeutic target for MYC-overexpressing liver tumors.

## Graphical Abstract

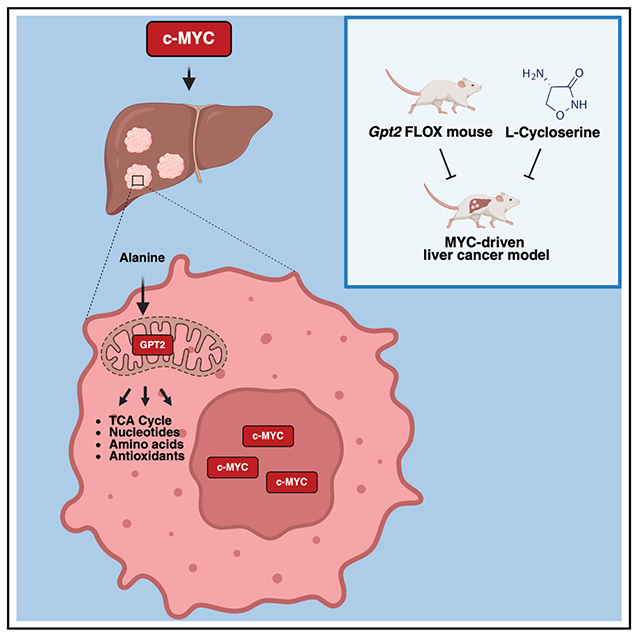

## INTRODUCTION

Liver cancer is a leading cause of cancer-related death, with over 750,000 mortalities annually worldwide.^1^ Combination immunotherapy regimens^2–4^ are the main treatments for surgically unresectable primary liver tumors. However, while a small subset of patients can experience deep and durable responses with combination immunotherapy, most patients progress and succumb to the disease, thus motivating our search for targeted therapies for this tumor type. Activation of MYC-dependent signaling is a pervasive event in liver cancer, and more than 30% of hepatocellular carcinoma (HCC) cases exhibit focal amplification or copy-number gain of the MYC locus.^5^ Since clinically relevant MYC inhibitors are lacking, we and others have sought to identify druggable pathways selectively essential for the survival of MYC-overexpressing cells.^6,7^ As a transcription factor, MYC can reprogram liver tumor metabolism by activating pathways such as glutaminolysis and glycolysis to meet the bioenergetic and biosynthetic requirements of cancer cells.^8^ We therefore hypothesized that identifying additional metabolic pathways selectively essential for MYC-dependent cancer cell growth (i.e., synthetic-lethal with MYC overexpression) could uncover therapeutic targets.

Alanine is the second most abundant amino acid in circulation, second only to glutamine, and can be used by the liver to generate glucose via gluconeogenesis, which is then secreted to fuel muscle contraction in a process termed the glucose-alanine cycle.^9^ Alterations in alanine metabolism are also implicated in diseased states; for instance, hepatic glutamic-pyruvic transaminase 2 (GPT2) levels increase in diabetes, leading to augmented gluconeogenesis and hyperglycemia.^10,11^ Key studies^12,13^ have also uncovered a role for tumor-microenvironment-derived alanine in driving Kras-driven pancreatic cancer and have described a role for alanine transporters in directing the flow of alanine from pancreatic stellate cells to tumor cells. However, the *in vivo* dependence on GPT2 and alanine metabolism in cancers originating from other tissues or driven by other oncogenes is not known. Whether MYC-driven liver cancers are reliant on alanine and if this pathway can be targeted with pharmacological inhibitors has not hitherto been reported.

Here, we describe a role for alanine catabolism in sustaining MYC-driven liver tumors. We found that MYC-driven liver cancer cells use alanine to promote their growth in a GPT2-dependent manner both in cell culture and in a mouse model of MYC-driven liver cancer. We uncovered that alanine was a substrate for numerous metabolic pathways important for cancer cell growth, including the tricarboxylic acid (TCA) cycle, non-essential amino acid synthesis, nucleotide biogenesis, and antioxidant production. Additionally, we found that L-cycloserine, an orally bioavailable compound that inhibits GPT2,^14^ slowed liver cancer cell growth both *in vitro* and *in vivo* in both transgenic mouse models and human liver cancer xenografts. Thus, GPT2-dependent alanine catabolism is exploited by MYC-driven liver tumors to enable their survival during nutrient deprivation, and inhibiting this pathway with L-cycloserine may be efficacious in patients with liver cancer.

## RESULTS

### MYC-driven liver tumors exhibit dependency on alanine metabolism

**Figure 1.**
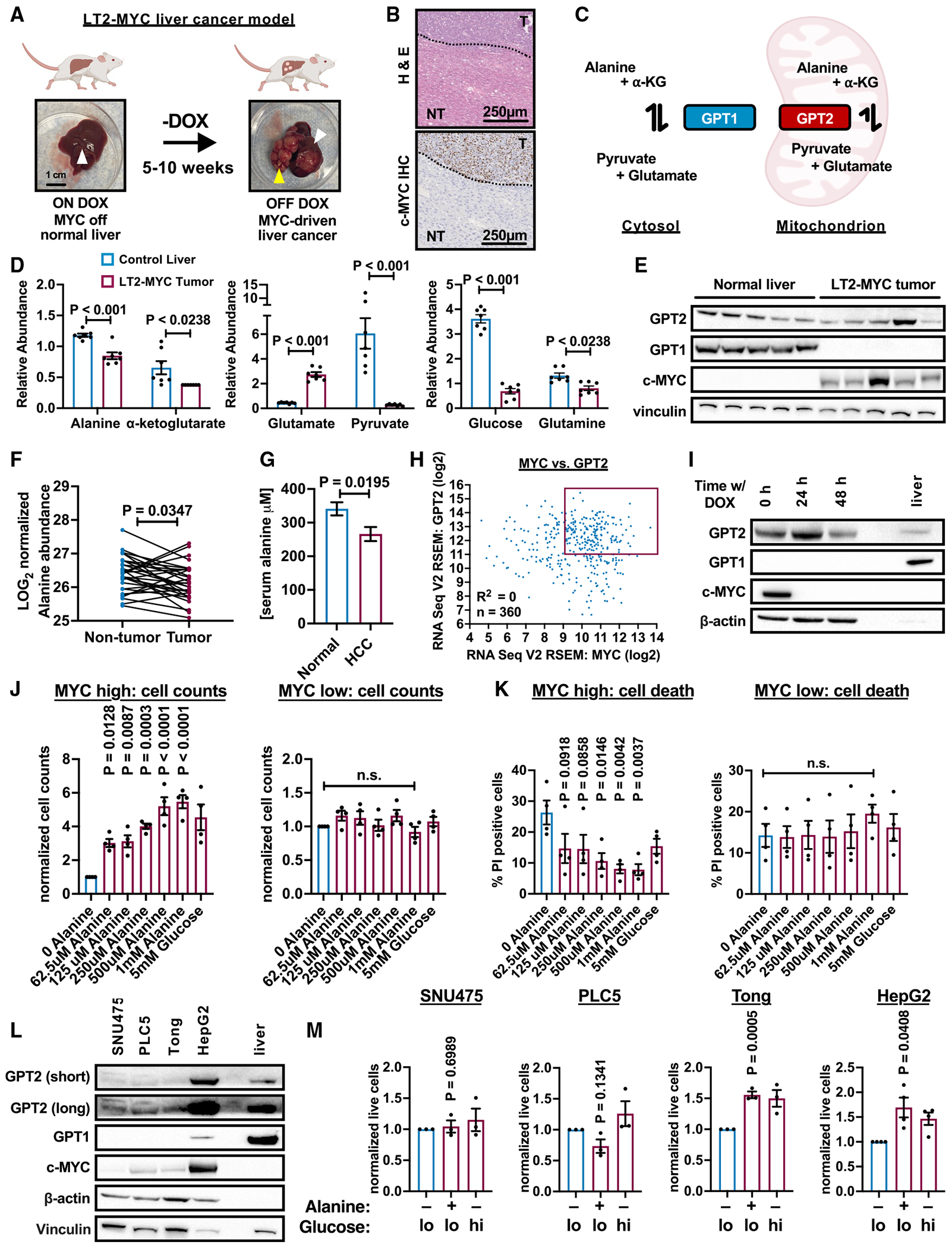
MYC-driven liver tumors exhibit a dependency on alanine metabolism (A) Schematic of the MYC regulatable liver cancer model (LT2-MYC). Representative images of LT2-MYC livers with and without doxycycline (DOX). White arrows indicate non-tumor liver tissue, and yellow arrows indicate tumors. Scale bar, 1 cm. (B) Tissue sections cut from formalin-fixed paraffin-embedded LT2-MYC tumors stained for hematoxylin and eosin or c-MYC (5× magnification). Dotted lines indicate tumor boundary. T, tumor; NT, non-tumor. Scale bar, 250 μm. (C) Diagram showing compartmentalized activity of GPT1 in the cytosol and GPT2 in the mitochondrion. (D) Log_2_ transformed abundance of glucose, glutamine, and alanine pathway metabolites in control liver (blue bars) versus LT2-MYC tumors (red bars) (*n* = 7 per group). (E) Immunoblotting of normal liver and LT2-MYC tumors for MYC and alanine enzymes. (F) Log_2_ transformed abundance of alanine by mass spectrometry in tumor and adjacent non-tumor tissue from human HCC (*n* = 30 per group), adapted from Budhu et al.^16^ (G) Serum alanine abundance in healthy patients (*n* = 10) and those with HCC (*n* = 14), from Watanabe et al.^17^ (H) Correlation of log_2_ transformed *Myc* and *Gpt2* transcript levels by RNA-seq in *n* = 360 human liver tumors, adapted from Ally et al.^5^ The red box indicates tumors that co-express the highest *Myc* and *Gpt2* mRNA. (I) Immunoblotting of alanine enzymes and MYC in EC4 cells treated with doxycycline (DOX) for 0–2 days to turn off MYC expression. Representative of three independent experiments. (J and K) Cell counts and cell death quantification in MYC-high (J) or MYC-low (K) EC4 cells cultured in indicated alanine levels with 500 μM glutamine (*n* = 4 independent experiments per condition). (L) Immunoblotting of MYC and alanine transaminase enzymes in a panel of human liver cancer lines. Representative of three independent experiments. (M) Proliferation response of a panel of human liver cancer lines cultured in 500 μM alanine (*n* = 3–4 independent experiments per line). Plots in (D), (G), (J), (K), and (M) show mean ± SEM. Plot in (F) shows alanine abundance in matched tumor and non-tumor tissue from individual HCC cases. Statistical significance determined using a two-sample *t* test in (D) and (G); matched pairs *t* test in (F); multiple one-sample *t* tests comparing alanine concentrations to control value of 1 with a Bonferroni correction for proliferation in (J) and (K); one-way ANOVA with Dunnett’s multiple-comparison test for cell death in (J) and (K); and one-sample *t* test in comparing alanine to control value of 1 in (M). See also Figure S1.

To identify unexplored metabolic pathways that could serve as therapeutic targets for HCC, we used MYC-driven liver tumors from the LAP-tTA;TRE-MYC (hereafter referred to as LT2-MYC) model, which exhibits features of aggressive HCC and hepatoblastomas.^15^ In this model, liver-specific MYC expression is induced when doxycycline is removed from the mouse diet (OFF DOX), leading to tumor formation in 5–10 weeks (Figure 1A). Tumors are distinguished by their cellular morphology and overexpression of MYC protein (Figure 1B). By mass spectrometry, we observed changes in metabolism related to the glutamic-pyruvic transaminase (GPT) isozymes (Figures 1C and 1D). GPT1 (cytosolic) and GPT2 (mitochondrial) catalyze the reversible transfer of an amino group from alanine to α-ketoglutarate, thus generating pyruvate and glutamate (Figure 1C). Notably, we observed that the substrates of GPT, alanine and α-ketoglutarate, were significantly reduced in LT2-MYC tumors compared to normal liver, whereas the product of GPT, glutamate, accumulated significantly in LT2-MYC tumors (Figure 1D). Pyruvate, the other product of GPT, was depleted, possibly by its consumption through other pathways (e.g., lactate dehydrogenase [LDH] or TCA cycle). Finally, glucose and glutamine, other metabolites used by MYC tumors to support their growth, were also depleted (Figure 1D), suggesting that MYC tumors might depend on the alanine metabolic pathway.

We compared protein expression levels of GPT enzymes in normal liver and LT2-MYC tumors by immunoblotting and found that normal liver expressed both the cytosolic (GPT1) and mitochondrial (GPT2) isoforms of GPT, whereas LT2-MYC tumors mainly expressed mitochondrial GPT2 (Figure 1E). This suggests that LT2-MYC tumors are dependent on mitochondrial alanine metabolism. Altogether, LT2-MYC tumors have lower alanine levels than normal liver tissue and specifically express mitochondrial GPT2, leading us to hypothesize that alanine was being catabolized by liver tumors to promote their growth and survival.

We then asked whether MYC directly or indirectly regulated GPT1/2 isozyme expression in tumors by leveraging data from a study^18^ that performed chromatin immunoprecipitation sequencing and RNA sequencing (RNA-seq) in the LT2-MYC model shortly after MYC induction. We observed that ODC1, a known direct MYC target gene,^19^ is upregulated in an MYC-dependent manner, thus validating this approach to identifying MYC target genes (Figure S1A). In contrast, we observed that GPT2 is neither a direct nor indirect transcriptional target of MYC (Figure S1B) and that GPT1 was downregulated in a MYC-independent manner (Figure S1A). We also tested whether MYC was indirectly promoting GPT2 expression in MYC tumors via induction of the unfolded protein response, as MYC has a well-documented role in activating translation of the transcription factor ATF4 in various tumor contexts via the induction of eIF2α phosphorylation,^20–22^ and ATF4 in turn is a direct transcriptional activator of GPT2.^10^ We interrogated the activity of this pathway by immunoblotting for ATF4 protein levels. However, we did not observe detectable ATF4 expression in MYC tumors as compared to control liver. Despite the increase in phosphorylated eIF2α observed in tumors (Figure S1B), the reduction in ATF4 protein indicated that GPT2 is not regulated by ATF4 in this tumor context. Together, our data do not support a role for MYC in regulating expression of GPT2 in LT2-MYC tumors, either through direct transcriptional control or indirect activation via unfolded protein response signaling.

We next investigated whether alanine was depleted in patients with liver tumors. Leveraging published liver cancer metabolomics datasets, we observed that alanine abundance was consistently decreased in human liver tumors as compared to non-tumor liver across multiple studies^16,23,24^ (Figure 1F). Moreover, a prior study^17^ examining amino acid levels in human liver cancer reported that alanine was depleted in the serum of patients with HCC compared to healthy subjects (Figure 1G), suggesting that augmented liver tumor alanine catabolism depleted it from the circulation. Finally, we investigated The Cancer Genome Atlas RNA-seq dataset^5^ of 360 samples from patients with HCC to understand the relationship between MYC and GPT2 in patients with liver cancer. We did not find a correlation between *MYC* and *GPT2* transcript levels (Figure 1H), consistent with our observations in the LT2-MYC model (Figure 1E). While MYC does not regulate GPT2 levels in human primary liver tumors, a majority of tumors co-express elevated *MYC* and *GPT2* transcripts, consistent with our transgenic mouse model (Figure 1H, upper right quadrant, box), which we propose is the subset of liver cancer patients in which alanine catabolism plays a functional role. Thus, the depletion of alanine in human serum, human liver tumors, and in a transgenic MYC-driven liver tumor model led us to further explore how alanine could be a metabolic substrate for liver cancer.

### Alanine promotes the proliferation of MYC-overexpressing liver cancer cells

Having observed that alanine depletion is conserved in both a mouse model and in human liver cancer, we used EC4 cells, a tumor line derived from the LT2-MYC model, to interrogate the role of alanine metabolism in cell culture. Similar to LT2-MYC tumors (Figure 1E), when EC4 cells were treated with doxycycline to shut off MYC expression, we observed that GPT2 was expressed, whereas GPT1 remained undetectable relative to normal liver lysates (Figure 1I). To test whether alanine is critical for the proliferation of EC4 cells, we cultured EC4 cells in minimal DMEM supplemented with 10% dialyzed fetal bovine serum (FBS) and 500 μM glucose and glutamine. We used these metabolite concentrations because they are comparable to the levels present in the serum of HCC patients.^17,25,26^ Alanine supplementation was sufficient to increase proliferation and viability in MYC-high EC4 cells (Figure 1J). In contrast, in EC4 cells that were pre-treated with doxycycline to turn off MYC expression, we did not observe appreciable changes in cell number or cell death after 4 days of culture in the same medium (Figure 1K), suggesting that alanine catabolism in EC4 cells is an MYC-dependent process. To further test whether MYC-low EC4 cells could utilize alanine for growth, we cultured them in even lower concentrations of glucose and glutamine (200–500 μM glucose and glutamine) and supplemented with 500 μM alanine. We did not observe a significant increase in growth with alanine supplementation at all glutamine and glucose levels tested (Figure S1C), suggesting that a nutrient-depleted environment is not in itself sufficient to render MYC-low EC4 cells responsive to alanine.

Next, we used a panel of human liver cancer lines with varying levels of GPT1/2 and MYC expression, which included the cell lines PLC5, SNU475, Tong, and HepG2, to test their dependence on alanine. Notably, PLC5 and SNU475, which express lower levels of MYC and GPT2 (Figure 1L), did not exhibit a difference in cell counts after 4 days of growth in minimal DMEM + 10% dialyzed FBS with 500 μM glucose and glutamine supplemented with 500 μM alanine (Figure 1M). In contrast, the HepG2 cell line, which expressed the highest levels of MYC and GPT2, showed increased cell proliferation when grown in the same medium supplemented with 500 μM alanine (Figures 1L and 1M). Notably, Tong cells, which expressed relatively lower MYC and GPT2 than HepG2, also showed increased cell proliferation in these cultured conditions (Figures 1L and 1M), suggesting that other factors might dictate alanine-dependent cell proliferation. We then titrated concentrations of glutamine and measured proliferation to better understand the parameters that dictate alanine responsiveness. We observed that alanine responsiveness depended on the concentration of glutamine present in cell culture, with HepG2 cells exhibiting the highest proliferative response when grown in 350 μM glutamine, and Tong cells showing the highest proliferative response in 400 μM glutamine (Figures S1D and S1E). Finally, we sought to test whether alanine-dependent cell proliferation in HepG2 cells was an MYC-dependent process. To accomplish this, we transfected HepG2 cells with pooled small interfering RNAs (siRNAs) targeting *MYC*, then seeded cells in medium containing 350 μM glutamine ± 500 μM alanine and measured cell counts after 4 days of growth. As expected, western blotting confirmed the efficacy of siRNA knockdown (Figures S1F and S1G). Moreover, we found that while HepG2 cells transfected with non-targeting siRNAs grew more rapidly with 500 μM alanine, *MYC* siRNA-transfected HepG2 cells did not grow more with alanine supplementation (Figure S1H), thus confirming that in HepG2 cells, proliferation supported by alanine catabolism is an MYC-dependent process. We therefore conclude that liver cancer cells from both mice and humans can utilize alanine to drive proliferation during glucose and glutamine limitation in an MYC-dependent manner.

### Circulating alanine is metabolized by MYC-driven liver tumors *in vivo*

**Figure 2.**
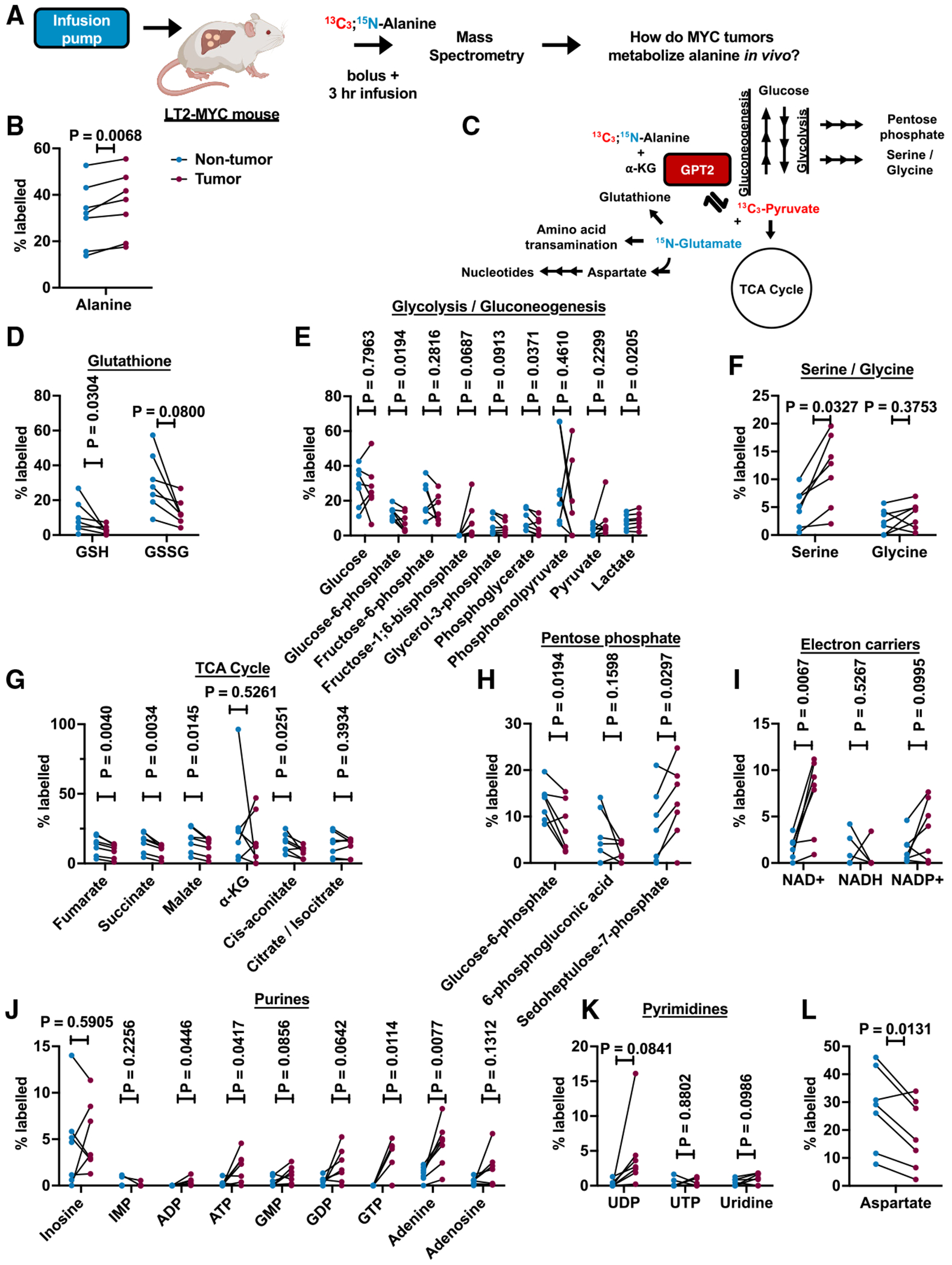
Circulating alanine is metabolized by MYC-driven liver tumors *in vivo* (A) Schematic showing the design of the *in vivo* isotope infusion study. (B) Contribution of ^13^C_3_, ^15^N-alanine to the alanine pool in tumor and non-tumor tissue. (C) Diagram of the GPT enzymatic reaction and pathways that were labeled by the alanine tracer. ^13^C^3^, ^15^N-alanine isotope labels are highlighted in red and blue, respectively. (D–L) Percentage of individual metabolites that had at least one label derived from ^13^C_3_,^15^N-alanine in pathways relating to glutathione (D), glycolysis and gluconeogenesis (E), serine-glycine synthesis (F), TCA cycle (G), electron carrier molecules (H), pentose phosphate pathway (I), purines (J), pyrimidines (K), and aspartate (L). *n* = 7 mice for each condition. Non-tumor tissue is indicated in blue and tumor tissue in red. Statistical significance in (B) and (D)–(L) determined using a matched-pairs *t* test comparing labeling percentage in non-tumor (blue) and tumor tissue (red) from the same animal. See also Figure S2.

Our finding that MYC-expressing liver cancer cells utilize alanine to drive their proliferation motivated us to investigate whether tumors use alanine as a fuel source. We therefore established an *in vivo* isotope-tracing strategy whereby tumor-bearing LT2-MYC mice were infused initially with a fully labeled ^13^C^3^,^15^N-alanine bolus and then continuously for 3 h (Figure 2A). Following infusion, tumor and non-tumor tissue were collected for pairwise comparison from each animal. The polar metabolites were extracted from each tissue and identified using liquid chromatography-mass spectrometry. We found that, on average, ~35% of the tumor alanine pool had at least one heavy isotope label (Figure 2B), thus confirming the efficacy of our infusion strategy. Notably, the ^13^C_3_,^15^N-alanine tracer made a greater contribution to the alanine pool in tumor as compared to non-tumor tissue (*p* = 0.0068), suggesting that MYC liver tumors might actively shuttle alanine to enable cancer cell catabolism. Having established and validated our *in vivo* tracing system, we next sought to understand the metabolic network downstream of alanine catabolism in MYC-driven liver tumors.

Tumor catabolism of alanine contributed to multiple metabolic pathways, including cellular bioenergetics, biosynthesis, and the oxidative stress response (Figure 2C). We observed diminished labeling of reduced glutathione in tumors compared to non-tumor tissue from the same animal (Figure 2D), consistent with our prior study^15^ showing that the expression of the rate-limiting glutathione enzyme GCLC is indirectly repressed by MYC in liver tumors. We also observed enhanced ^13^C labeling of lactate by alanine in tumors (Figure 2E), consistent with the well-established role of MYC in promoting transcription of LDH-A.^27^ Finally, we observed that the labeling of serine was significantly higher in tumor tissue than in non-tumor tissue (Figure 2F), consistent with prior work showing that serine synthesis is an MYC-stimulated process in HCC.^28^ Thus, our isotope-tracing approach demonstrates that alanine contributes to known MYC-regulated metabolic pathways *in vivo*.

The contributions by alanine to other metabolic pathways are less understood. In terms of how alanine contributes to tumor bioenergetic pathways, we observed that alanine-derived ^13^C labeled between 10% and 20% of the pool of TCA-cycle intermediates in both tumor and non-tumor tissue (Figure 2G), confirming that alanine-derived ^13^C-pyruvate contributes to TCA-cycle anaplerosis. Moreover, alanine labeling of the electron carrier NAD^+^ was higher in MYC tumors (Figure 2H), an additional mechanism by which alanine supports bioenergetics. Finally, we also observed that the labeling of energy carriers ATP and GTP was higher in tumor tissue compared to non-tumor tissue (Figure 2J). Thus, we find that alanine contributes to MYC-driven tumor bioenergetics in a multifaceted manner.

Given that alanine can be converted to pyruvate, which serves as a substrate for gluconeogenesis in normal liver,^9^ we sought to understand how this pathway is altered in liver tumors. We observed no significant differences in ^13^C-pyruvate labeling by ^13^C_3_,^15^N-alanine in tumors and non-tumor tissue (Figure 2E). These data suggest that MYC-driven liver tumors maintain the ability to convert alanine to pyruvate for gluconeogenesis. We then examined individual gluconeogenic intermediates and observed a reduction in ^13^C labeling of glucose-6-phosphate in tumors (Figure 2E), possibly due to increased conversion into intermediates of the pentose phosphate pathway, which is augmented in liver tumors.^29^ Additionally, we observed reduced ^13^C labeling of phosphoglycerate in tumor tissue (Figure 2E), possibly due to enhanced tumor serine synthesis, which utilizes phosphoglycerate as a substrate.^28^ Unexpectedly, we also observed that ^13^C-glucose labeling from alanine-derived pyruvate did not differ between tumor and non-tumor tissue, suggesting that MYC tumors retain a fully intact gluconeogenesis pathway. This is notable, given that the rate-limiting gluconeogenesis enzymes FBP and G6PC1 are generally downregulated in liver tumors,^30–32^ a trend that we also observed in the LT2-MYC tumor model (Figure S2A, adapted from Kress et al.^18^). This suggests that while gluconeogenesis is indeed repressed at the transcript level, residual enzymatic activity is still sufficient to sustain pathway activity in MYC tumors *in vivo*. Thus, we find that alanine supports gluconeogenesis in MYC tumors.

Given that pyruvate and glutamate, the products of GPT2, contribute to biosynthesis, we next asked whether alanine contributes to biosynthetic pathways in MYC tumors. We observed that alanine contributed to the production of most amino acids (Figure S2D), consistent with the role of glutamate as an amino donor in transamination reactions.^33^ Specifically, we observed that serine and sarcosine had higher labeling in tumors, whereas aspartate, arginine, glutamine, glutamate, and tyrosine had lower labeling in tumors (Figures S2D and 2L). Multiple products of purine synthesis were also labeled at a higher efficiency in MYC tumors, specifically ADP, ATP, GTP, and adenine (Figure 2J). Notably, multiple alanine-derived metabolites, including glutamine, glycine, and aspartate, as well as ATP, directly feed into purine synthesis, suggesting multiple mechanisms by which alanine supports this pathway.^33^ We also observed a contribution of alanine to the pentose phosphate pathway, which generates ribulose-5-phosphate, a precursor of purine synthesis.^34^ Specifically, in tumors we observed higher labeling of the sedoheptulose-7-phosphate and reduced labeling of glucose 6-phosphate (Figure 2I), the glycolytic intermediate from which the pentose phosphate pathway branches off, suggesting that alanine might coordinate pentose phosphate pathway activity and purine biogenesis. Alanine also made a lesser contribution to the production of pyrimidines than to purines, with similar labeling efficiency (1%–5%) of UDP, UTP, and uridine in tumor and non-tumor tissue (Figure 2K). Finally, we also observed labeling of metabolites relating to polyamines, hexosamines, and the urea cycle in both tumor and non-tumor tissue (Figures S2B, S2C, and S2E). In summary, our *in vivo* tracing studies provide direct evidence confirming that MYC liver tumors consume alanine in circulation and catabolize it to support tumor energy production, biosynthesis, and glutathione production.

### MYC-driven liver tumorigenesis is dependent on GPT2 expression

**Figure 3.**
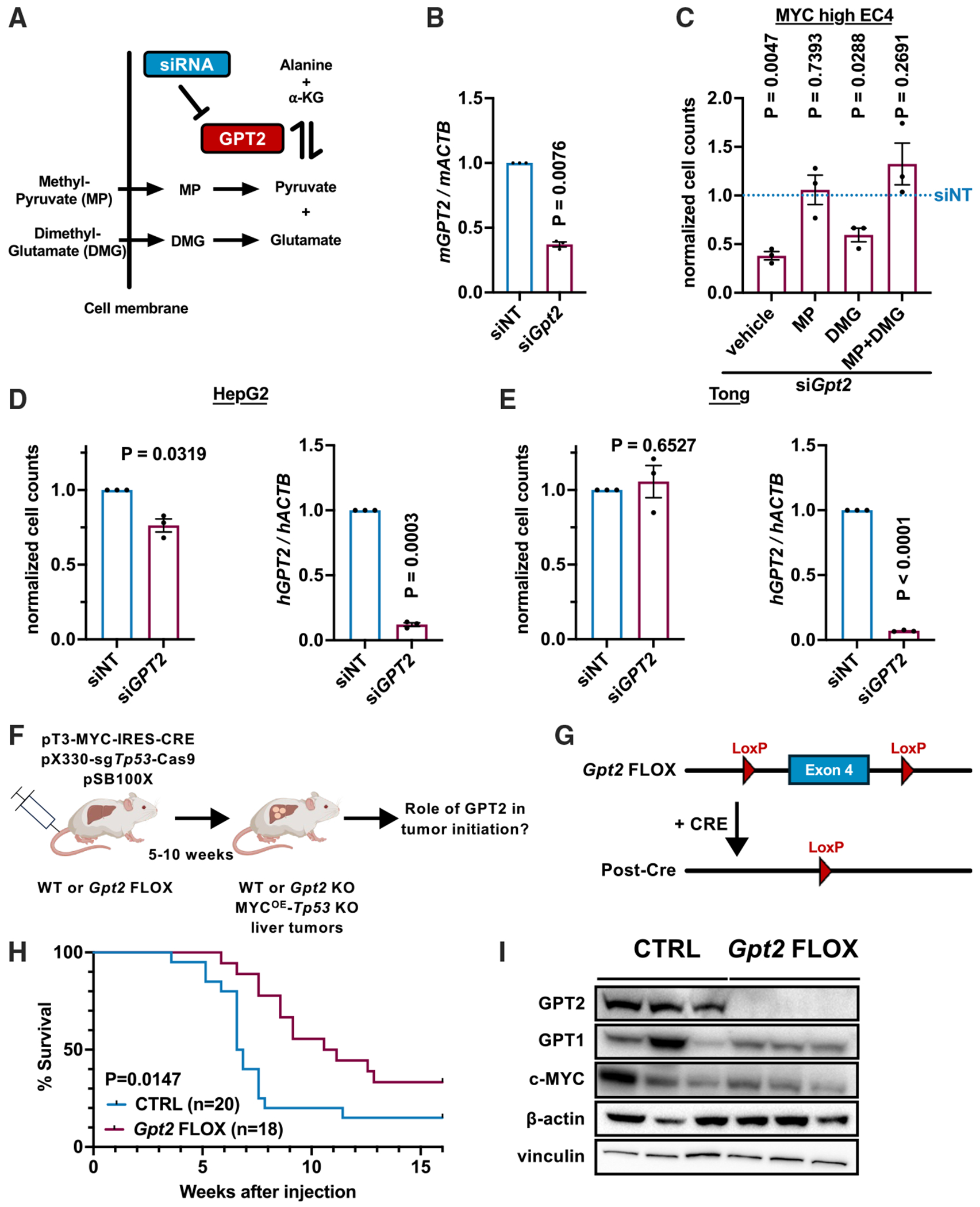
MYC-driven liver tumorigenesis is dependent on GPT2 expression (A) Diagram of siGPT2 metabolite rescue study. (B) Quantification of *Gpt2* transcript levels in MYC-high EC4 cells by qPCR (cDNA from *n* = 3 independent experiments). (C) Quantification of final cell number after 96 h with or without siRNA against *Gpt2* in MYC-high EC4 cells cultured in 500 μM alanine medium upon treatment with 500 μM methylpyruvate and/or dimethylglutamate (*n* = 3 independent experiments each group). Samples were normalized to the cell counts of control siNT cells (blue dotted line). (D) Proliferation response of HepG2 cells to *GPT2* knockdown in 500 μM alanine medium, and qPCR knockdown validation of *GPT2* knockdown (*n* = 3 independent experiments). Cell counts and transcript levels of non-targeting control were both set to 1. (E) Proliferation response of Tong cells to *GPT2* knockdown in 500 μM alanine medium, and qPCR knockdown validation of *GPT2* knockdown (*n* = 3 independent experiments). Cell count and transcript levels of non-targeting control were both set to 1. (F) Diagram of hydrodynamic tail vein transfection in mouse liver for tumor initiation study. (G) Schematic showing how CRE deletes exon 4 of *Gpt2*. (H) Survival of wild-type (*n* = 20) versus *Gpt2* FLOX (*n* = 18) mice upon liver tumor induction. (I) Immunoblotting of wild-type and GPT2 knockout tumors. Plots in (B)–(E) show mean ± SEM of normalized cell counts or normalized transcript levels where indicated. Statistical significance determined using multiple one-sample *t* tests with Bonferroni correction with the non-targeting control set to 1 in (B); one-sample *t* test with the non-targeting control set to 1 in (C)–(E); and log-rank test in (H). See also Figure S3.

We next sought to complement our descriptive isotope-tracing studies with loss-of-function experiments to understand the mechanistic basis of alanine catabolism. Given that MYC-overexpressing cells have low levels of GPT1, we hypothesized that these cells are reliant on GPT2 for alanine-dependent metabolites that support cell growth in limiting glucose and glutamine conditions. To test this, we depleted *Gpt2* in MYC-high EC4 cells using *Gpt2*-targeting or non-targeting siRNAs as a control (siNT) before measuring total cell number after culturing the cells under limiting conditions of glucose and glutamine, supplemented with 500 μM alanine, for 4 days. We then supplemented with the cell-permeable analogs of pyruvate (methylpyruvate) and glutamate (dimethylglutamate) at 500 μM to test whether these metabolites, which are the products of the GPT2 reaction, can rescue cell proliferation in the absence of GPT2 (Figure 3A). As expected, *Gpt2* transcript levels were reduced upon siRNA knockdown by quantitative PCR (qPCR), and we observed a significant reduction in total cell counts in the vehicle-treated *Gpt2* knockdown condition as compared to siNT controls (Figures 3B and 3C). Moreover, methylpyruvate restored si*Gpt2* cell numbers to the levels of the siNT control, whereas dimethylglutamate treatment alone was insufficient (Figure 3C), demonstrating that MYC cells depend on GPT2-derived pyruvate. In contrast, in MYC-low EC4 cells, we observed a smaller effect on cell counts upon *Gpt2* knockdown (~30% versus ~55% reduction in MYC-low versus MYC-high; Figures S3A and S3B), confirming that MYC expression is a key determinant of sensitivity to *Gpt2* knockdown. Finally, in human liver tumor cell lines, we observed a reduction in total cell number upon *GPT2* knockdown in HepG2 cells but not in Tong cells (Figures 3D and 3E). Knockdown efficacy was also confirmed by qPCR in both lines (Figures 3D and 3E). Notably, Tong cells exhibit lower MYC and GPT2 expression compared to HepG2 cells, yet they still utilize alanine for proliferation (Figure 1M); this suggests that alanine can promote the proliferation of human liver tumor cell lines through both GPT2-dependent and -independent mechanisms. Thus, our studies identify a dependence on GPT2 in alanine-driven cell growth in cells derived from the LT2-MYC model and in human liver cancer cells with high MYC expression.

We next sought to test the requirement for GPT2 in the development of primary liver cancer. Toward this goal, we generated a transposon construct that allowed for the simultaneous expression of MYC and CRE recombinase, which we designated pT3-MYC-IRES-CRE. Either wild-type mice or mice that had a *Gpt2* allele in which the 4th exon was flanked by *LoxP* motifs (*Gpt2* FLOX^10^) had tumor formation induced by somatic transduction of oncogenes via hydrodynamic tail vein injection. Mice received pT3-MYC-IRES-CRE, together with a construct to delete *Tp53* (pX330-Cas9-sg*Tp53*)^35^ and the sleeping beauty transposase (pSB100X),^36^ to generate tumors (Figures 3F and 3G).^37^ Thus, these tumors are driven by both MYC overexpression and *Tp53* loss and harbor either intact or deleted *Gpt2*. We observed a marked difference in tumor burden in mice in which *Gpt2* was deleted; mice with *Gpt2* deletion also demonstrated a significant increase in median survival from 6.7 to 10.9 weeks compared to control mice (Figure 3H). Notably, tumor penetrance also decreased from 85% to 66% in control versus *Gpt2* FLOX mice (Figure 3H). We then profiled protein expression in tumors generated in control and *Gpt2* FLOX mice and confirmed MYC overexpression and complete loss of GPT2 expression; we also failed to observe compensatory upregulation of GPT1 in GPT2-null tumors (Figure 3I). Thus, our studies demonstrate an *in vivo* functional role for GPT2 in MYC-driven liver tumor initiation.

### L-Cycloserine as a therapy for MYC-driven liver cancer

**Figure 4.**
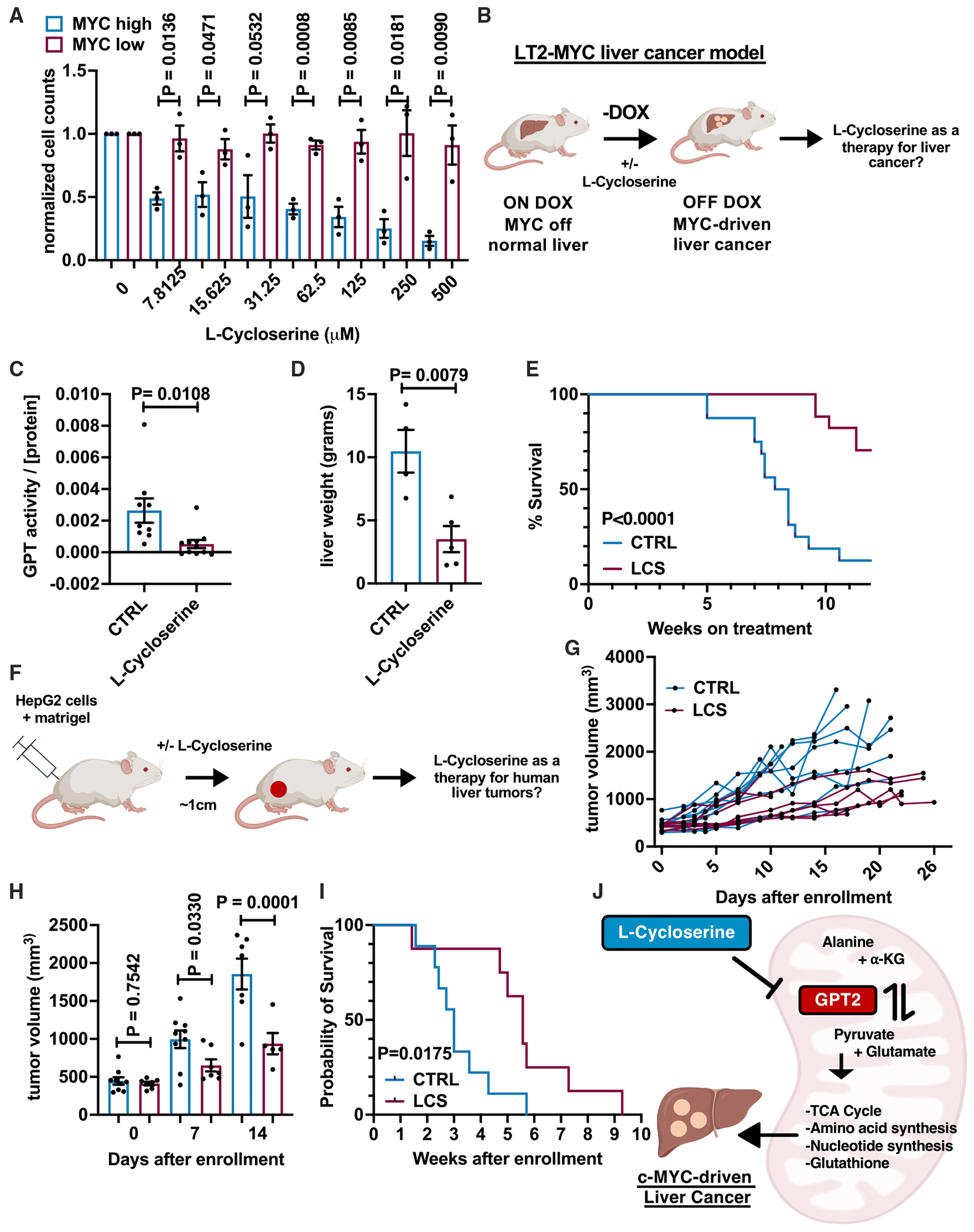
L-Cycloserine as a therapy for MYC-driven liver tumors (A) Quantification of MYC-high (blue) or MYC-low (red) EC4 cell counts in 500 μM alanine/low glutamine medium upon treatment with indicated L-cycloserine levels, as assayed by propidium iodide and Hoechst staining, with untreated control cells set to 1 (*n* = 3 independent experiments for each condition). (B) Diagram describing the L-cycloserine tumor initiation study in the LT2-MYC model. (C) Quantification of *in vitro* GPT enzymatic activity in LT2-MYC tumors treated with 500 mg/L L-cycloserine until ethical endpoint (*n* = 9 control [CTRL] and *n* = 11 cycloserine tumors per group). (D) Tumor weights of LT2-MYC mice after 8 weeks of treatment with 500 mg/L L-cycloserine in drinking water (*n* = 4 CTRL and *n* = 5 L-cycloserine mice). (E) Survival of LT2-MYC mice treated with or without 500 mg/L L-cycloserine from the onset of MYC induction (*n* = 16 CTRL and *n* = 17 L-cycloserine mice). (F) Diagram describing the L-cycloserine HepG2 tumor maintenance study. (G) Individual tumor volumes of HepG2 tumor xenografts after treatment with 500 mg/L L-cycloserine or water control. (H) Average tumor volumes of HepG2 tumor xenografts after treatment with 500 mg/L L-cycloserine (*n* = 7 mice; red bars) or water control (*n* = 9 mice; blue bars). (I) Survival comparison of HepG2 xenograft-bearing mice treated with or without L-cycloserine. Endpoint was when tumors reached 2 cm in diameter. (J) Summary of the role of GPT2 in MYC-driven liver tumorigenesis. Plots in (A), (C), (D), and (H) show mean ± SEM. Statistical significance determined using two-sample *t* test in (A), with Holm-Sidak multiple-comparisons correction in (H); two-sample *t* test in (C) and (D); and log-rank test in (E) and (I). See also Figure S4.

Our loss-of-function GPT2 studies suggest that drugs inhibiting GPT2 are potentially useful for slowing or preventing liver cancer growth. We therefore investigated the utility of inhibiting GPT2 with the compound L-cycloserine. While L-cycloserine is not a specific inhibitor of GPT2 and may also inhibit the enzymes aspartate transaminase (AST) and serine palmitoyltransferase (SPT) at higher concentrations,^14,38,39^ its oral bioavailability and ability to inhibit GPT2 motivated us to undertake preclinical studies to examine its potential as a therapeutic for MYC-driven liver cancer. To test this concept, we first used an *in vitro* system wherein EC4 cells with high or low MYC expression were treated with increasing levels of L-cycloserine. We observed that after 4 days of growth, MYC-high EC4 cells exhibited a dose-dependent reduction in cell counts with increasing L-cycloserine concentration, whereas the proliferation of MYC-low EC4 cells was not inhibited to the same extent (Figure 4A). Given these data, we next investigated the *in vivo* efficacy of this compound in LT2-MYC tumor development. MYC expression was induced in a cohort of LT2-MYC mice at weaning by removing doxycycline from their diet, and mice received water with 500 mg/L of L-cycloserine to block GPT2 activity (Figure 4B). We used a fluorometric enzymatic assay to quantify GPT1/2 activity in the liver tumors of mice treated with L-cycloserine and confirmed that enzymatic activity was effectively abolished in drug-treated mice (Figure 4C). We also tested the efficacy of L-cycloserine as a therapeutic by treating a cohort of mice in this manner for 8 weeks and sacrificed the mice to assess tumor burden. We observed a pronounced difference in tumor burden as measured by liver mass, with vehicle-treated mice having an average liver weight over twice that of L-cycloserine-treated animals (Figure 4D). Finally, we repeated the study to examine the effects on survival and found that L-cycloserine improved median time to ethical endpoint and markedly reduced tumor incidence from ~90% in the vehicle group to 30% in the drug-treated group (Figures 4E and S4A). Thus, L-cycloserine treatment reduces tumor initiation and burden in a mouse model of MYC-driven liver cancer.

Based on the observation that L-cycloserine was an effective therapy in a mouse model of liver cancer, we next sought to test the efficacy of L-cycloserine in established human liver tumors. We generated mice bearing HepG2 xenografts, a context in which both MYC and GPT2 are highly expressed (Figure 1M), and treated mice with L-cycloserine once tumors reached 1 cm in length (Figure 4F). We observed that the tumor volume of L-cycloserine-treated mice was markedly diminished as compared to control tumors (Figures 4G and 4H) and that L-cycloserine treatment extended the time for tumors to reach the ethical endpoint (2 cm in maximal diameter; Figure 4I). Thus, these preclinical studies provide rationale for using L-cycloserine and developing more selective GPT2 inhibitors as therapeutics in patients with MYC-driven liver tumors.

## DISCUSSION

A cure for liver cancer remains a major unmet public health need, and MYC overexpression demarcates an aggressive and difficult-to-treat subset of tumors.^1,40,41^ Our work expands our understanding of how MYC reprograms tumor metabolism and identifies a potential therapeutic approach for treating MYC-high liver cancers.

Our studies reveal that alanine catabolism in liver cancer differs from previous findings with respect to tissue and oncogene contexts. Compared to Kras-driven pancreatic cancer, wherein stromal cells undergo autophagy and secrete alanine into the microenvironment,^12,13^ we find that MYC-driven liver tumors take up and catabolize alanine from the circulation. Additionally, our work demonstrates that alanine catabolism is a druggable process, providing a rationale for repurposing L-cycloserine or other clinically relevant GPT2 inhibitors as a therapy for tumors that catabolize alanine.

These findings also suggest that MYC coordinates the generation and usage of multiple pools of pyruvate to ensure a balance between glycolysis and TCA-cycle activity. MYC-dependent regulation of glucose transporters and glycolytic enzyme expression drives augmented glucose catabolism and lactate generation from pyruvate in the cytosol. This supports biosynthesis via shunting of glycolytic intermediates and regenerates NAD^+^ to maintain high rates of glycolysis.^42,43^ In contrast, the generation of pyruvate via the mitochondrial-localized GPT2 enzyme^44,45^ might preferentially allow the usage of pyruvate by mitochondrial pathways such as the TCA cycle. Thus, MYC’s co-ordination of compartmentalized pyruvate generation could permit the simultaneous activity of the TCA cycle and aerobic glycolysis without the two processes having to compete for a limited supply of pyruvate.

Future studies in this area should examine how inhibition of alanine catabolism functionally interacts with other cancer therapeutics. For instance, T cells are alanine auxotrophs due to their lack of GPT1/2 expression and depend on extracellular alanine to sustain protein synthesis and T cell activation.^46^ As a result, inhibiting tumor cell alanine catabolism with L-cycloserine could augment tumor microenvironmental levels of alanine, thus potentially promoting T cell activation and enhancing the efficacy of immune checkpoint blockade. Another open question concerns the identity of the compensatory pathways that enable tumor survival in the context of GPT2 inhibition. Pathways that generate the same metabolites as GPT2, for instance glutaminase^47^ or other transaminases, could functionally compensate for GPT2 inhibition and may also be suitable targets for combinatorial metabolic therapy strategies.

It remains an open question why MYC tumors use alanine as a metabolic substrate. Notably, we did not observe evidence for a gene-regulatory link between MYC and GPT2 in the LT2-MYC model or in human liver tumors (Figures 1H and S1A), although many human liver cancers express both genes at high levels (Figure 1H, red box). It is possible that highly proliferative and metabolically active liver tumors are more likely to utilize alanine to drive their growth, regardless of their driver oncogene, due to the high levels of GPT2 present in this tissue context. Notably, key studies have also shown that tumors with identical oncogenic drivers (i.e., MYC) arising in different tissue contexts can have drastically different metabolic dependencies, suggesting that the metabolic profile of a tumor is influenced by the tissue context in which it develops.^42,48^ It is also possible that tumors arising in other tissue contexts with high levels of GPT isozyme expression are more likely to leverage alanine as a metabolic substrate to support their proliferation. Further work will be required to delineate the contributions offered by the tissue of origin, the oncogenic profile, and the proliferative rate of a tumor in determining its ultimate metabolic phenotype.

Finally, while we observed efficacy of the compound L-cycloserine *in vivo*, caution should be used in interpreting these results. L-Cycloserine is not a specific GPT2 inhibitor and inhibits AST and SPT at higher concentrations.^14,38,39^ Thus, the antitumor properties of L-cycloserine may be due in part to inhibition of other enzymes. Nevertheless, our studies demonstrate the utility of targeting GPT2 in liver tumors with both genetic and chemical approaches. The data presented here provide strong rationale for discovering more potent and specific GPT2 inhibitors that may prove to be useful as tools for research or as therapy in the clinic.

Collectively, our work supports a model (Figure 4J) wherein GPT2-dependent alanine catabolism supports MYC-driven liver cancer by fueling tumor bioenergetics and supporting biomass generation. Our results also argue that inhibiting alanine catabolism with the compound L-cycloserine, or other inhibitors of GPT2, could be an effective therapy for this aggressive and intransigent subset of liver cancer.

### Limitations of the study

In this study, we investigated the role of GPT2-dependent alanine catabolism in a panel of human liver cancer lines. Notably, only HepG2 cells exhibited a dependency on GPT2-dependent alanine catabolism, while the remaining human lines were either alanine insensitive (SNU475 and PLC5) or alanine responsive but not GPT2 dependent (Tong). It may be worthwhile to interrogate the role of the alanine-glyoxylate aminotransferase (AGXT) isozymes or the catabolism-independent function of alanine in these GPT2-independent cellular contexts. Furthermore, our HepG2 L-cycloserine tumor maintenance studies do not specifically test the role of GPT2 in this context, given the off-target effects of L-cycloserine, which we previously discussed. Therefore, it remains an open question as to whether GPT2 is required for the growth of established liver tumors. Finally, as the cell lines and mice used for the studies were all male, the generalizability of our findings might be limited by this aspect.

## RESOURCE AVAILABILITY

### Lead contact

General inquiries should be addressed to the lead contact, Andrei Goga (andrei.goga@ucsf.edu).

### Materials availability

All materials requests should be addressed to the lead contact. The pT3-MYC-IRES-CRE vector will be deposited at Addgene.

## STAR★METHODS

### EXPERIMENTAL MODEL AND STUDY PARTICIPANT DETAILS

#### Conditional transgenic model of MYC-driven liver cancer

All mouse work was approved by UCSF Institutional Animal Care and Use Committee (IACUC) under protocol number AN200579. All mice were housed and bred in pathogen-free conditions at 23°C under a 12-h light: 12-h dark cycle in the Animal Barrier Facility at UCSF. Animals were regularly examined by a veterinarian for general health.

The LAP-tTA; TetO-cMYC; FVBN (LT2-MYC) mouse model has been previously described by Shachaf et al.^51^ LT2-MYC breeders were maintained on doxycycline (dox) chow to repress oncogene expression (200 mg/kg, Bio-Serv, *ad libitum* feeding) and male pups were taken off dox chow at weaning to turn on MYC expression and induce tumor formation. Male mice were used because the TetO-cMYC transgene is on the Y chromosome. Tumors typically form within 5–10 weeks, and ethical endpoint was determined by body score and abdominal palpation. Tumor samples were collected in OCT (Fisher Healthcare, 4585), 4% PFA in DPBS (Electron Microscopy Services, 15710), or snap frozen in liquid nitrogen.

For the L-cycloserine tumor initiation study, male LT2-MYC mice were taken off dox chow at weaning and littermates were allocated to either be treated with normal water (control) or water with 500 mg/L L-cycloserine (MedChemExpress, HY-B1122). Mice were monitored for tumor formation and sacrificed at ethical endpoint as determined by body condition score.

#### Hydrodynamic model of MYC-driven liver cancer

Hydrodynamic tail vein injection was done on control wildtype male C57BL/6 (JAX, 000664) or *Gpt2* FLOX (10) mice of 6–8 weeks of age as done by Ruiz De Galarreta et al.^37^ For every 2 mL of normal saline (Intermountain Life Sciences, Z1377), 13 μg of pT3-EF1-MYC-IRES-CRE, 13 μg px330-p53 (Addgene, 92046) and 520 ng of the pSB100X (Addgene, 34879) plasmids were dissolved and the solution then filtered through a .22 μm filter (Fisherbrand, 09-720-511) and used the same day. Mice were injected using 27.5 G butterfly needles (Terumo, SV*27EL) with a volume of plasmid solution equal to 10% of the mouse’s body weight in 5–8 s. Tumors formed within 5–10 weeks, and ethical endpoint was determined by body score and abdominal palpation. Tumor samples were collected in optimal cutting temperature (OCT), 4% PFA, or snap frozen. Mice imported from Jackson Labs were allowed a week to acclimate to the animal facilities before being used for the experiments.

#### Xenograft model of liver cancer

HepG2 xenografts were established by injecting cells in Matrigel (Corning, 356234) into the hind flank of male nude mice (JAX, 002019) 6–8 weeks of age. HepG2 cells were lifted at 75–90% confluency and resuspended at a concentration of 20 × 10^6^ cells/mL in DPBS (Gibco, 14190-144), and each mouse was injected with 100 μL of a 1:1 mix of Matrigel and cell suspension in DPBS using 25 5/8G syringes (BD, 309626). Tumor onset was monitored by caliper measurement of subcutaneous tumors. For tumor maintenance studies, once tumor length (L) reached 1 cm, mice were allocated to either be treated with normal water (control) or water with 500 mg/L L-cycloserine which was replaced weekly. Mice were sacrificed once tumors reached 2 cm in length, and tumors were collected in OCT, 4% PFA, or snap frozen. Tumor volume was calculated using the formula V= (L*W^2^)/2. Mice imported from Jackson Labs were allowed a week to acclimate to the animal facilities before being used for the experiments.

#### Cell culture

All cell lines were maintained at 37°C and 5% CO_2_ and cultured in high glucose DMEM (Gibco, 11995-065) supplemented with 10% FBS (Gibco, 10437-028) and 100 μM non-essential amino acids, (NEAA) (UCSF Cell Culture Facility, CCFGA001-22BU01) and were passaged every 2–3 days. EC4 cells were a gift from Dean Felsher (Stanford) and were derived from a tumor-bearing male LT2-MYC mouse. HepG2 (HB-8065) cells were purchased from ATCC. The PLC5 (CRL-8024), SNU475 (CRL-2236), and Tong (CVCL_V640) cell lines were a gift from Xin Chen (University of Hawai’i Cancer Center). All human cell lines used in this study were derived from the liver tumors of male patients.

All cell lines tested negative for mycoplasma, and human cell lines were validated by STR profiling (U Arizona Genetics Core).

### METHOD DETAILS

#### Experimental design

For animal studies the replicates represent the number of animals in each treatment group. For *in vitro* cell proliferation assays, the replicates represent the number of independent experiments performed. For *in vitro* enzymatic assays (Figure 4C), the replicates represent the number of tumor samples used for each group. The exact number of replicates, *n*, for each experiment is described in the figure legends. There was no strategy for randomization or sample size determination, and the investigator was not blinded. For the HepG2 xenograft study (Figures 4F–4I), mice where xenografts grew as multiple separate tumors were not enrolled in the study. Otherwise, no mice were excluded from the animal studies.

#### Transposon for transduction of oncogenes

The pT3-MYC-IRES-CRE vector was synthesized by GeneScript. The plasmid sequence was identical to that of the pT3-EF1a-MYC-IRES-LUC plasmid (Addgene, 129775), except the two *LoxP* sites were deleted, and the Luciferase coding sequence was replaced with the Cre coding sequence. Plasmids were propagated in NEB stable competent cells to prevent recombination.

#### Histology and immunohistochemistry

Mice were sacrificed according to institutional protocols, then perfused with DPBS and tissues collected and fixed overnight in 4% paraformaldehyde (Electron Microscopy Sciences, #15700) on an orbital shaker. Tissues were then placed in 70% ethanol, and processed, stained, and imaged by Histowiz for c-MYC (Abcam, ab32072) and Hematoxylin and Eosin.

#### Metabolomics

Steady state metabolomics of control livers and LT2-MYC tumors was performed and analyzed by Metabolon, as reported in Anderton et al.^15^

*In vivo* isotope tracing was performed on LT2-MYC mice that had been off DOX chow for 8–10 weeks. Tumor-bearing mice were anesthetized by isoflurane and kept on heating pads. Mice then had a catheter (Instech, C20PU-MJV2014) inserted into their jugular vein and tied in place using sutures (Surgical Specialties, SP117) and the catheter was then flushed with 50 μL heparin (McKesson, NDC 63739-931-14) to prevent coagulation. Mice were then infused with a ^13^C_3_; ^15^N-alanine (Cambridge Isotopes, CNLM-534-H-PK) bolus of 0.114 mg per g body weight, followed by an infusion of 0.003 mg per g body weight per minute for 3 h at a flow rate of 0.15 mL per hour using a Pump 11 Elite Dual Syringe Infusion Pump (Harvard apparatus, 70–4501). ^13^C_3_; ^15^N-alanine was dissolved in sterile normal saline at 48 mg/mL. After infusion, mice were sacrificed, perfused with saline, and tumor and non-tumor tissue dissected and snap frozen.

Polar metabolite extraction from tissue samples was done by using a sharp blade to cut off a small piece of tissue 10–30 mg in weight. Each sample was placed in a 2 mL tube with a 5 mm stainless-steel bead (Qiagen, 69989) and 1 mL of methanol extraction solution composed of 80% methanol: 20% H_2_O with 100 nM trifluoromethanesulfonate (Fisher, A456-500; Fisher, W6-212; Sigma, 422843-5G). Samples were then extracted on a TissueLyser LT (Qiagen, 85600) by running samples for 1 min at max speed, followed by a 1 min break, three consecutive times. After extraction, samples were rested at −20°C for 10 min, and then vortexed and centrifuged at 17,000 x g for 10 min at 4°C. 700 μL of the supernatant was then transferred to new tubes and centrifuged in the same manner again. The top 500 μL of the supernatant was then transferred into a new tube and a volume of sample corresponding to a tissue equivalent of 4 mg was transferred into a new tube for evaporation. Samples were run on a Savant DNA120 SpeedVac System (ThermoFisher, DNA120-115) on high for 1–2 h, or until the sample was completely evaporated. Samples were then stored at −80°C until they were run on the LC/MS.

Dried metabolites were reconstituted in 100 μL of a 50% acetonitrile (ACN) 50% dH20 solution. Samples were vortexed and spun down for 10 min at 17,000g. 70 μL of the supernatant was then transferred to HPLC glass vials. 10 μL of these metabolite solutions were injected per analysis. Samples were run on a Vanquish (Thermo Scientific) UHPLC system with mobile phase A (20mM ammonium carbonate, pH 9.7) and mobile phase B (100% ACN) at a flow rate of 150 μL/min on a SeQuant ZIC-pHILIC Polymeric column (2.1 × 150 mm 5 μm, EMD Millipore) at 35°C. Separation was achieved with a linear gradient from 20% A to 80% A in 20 min followed by a linear gradient from 80% A to 20% A from 20 min to 20.5 min 20% A was then held from 20.5 min to 28 min. The UHPLC was coupled to a Q-Exactive (Thermo Scientific) mass analyzer running in polarity switching mode with spray-voltage = 3.2kV, sheathgas = 40, aux-gas = 15, sweep-gas = 1, aux-gas-temp = 350°C, and capillary-temp = 275°C. For both polarities mass scan settings were kept at full-scan-range = (70–1000), ms1-resolution = 70,000, max-injection-time = 250ms, and AGC-target = 1E6. MS2 data was also collected from the top three most abundant singly-charged ions in each scan with normalized-collision-energy = 35. Each of the resulting “.RAW” files was then centroided and converted into two “.mzXML” files (one for positive scans and one for negative scans) using msconvert from ProteoWizard. These “.mzXML” files were imported into the MZmine 2 software package. Ion chromatograms were generated from MS1 spectra via the built-in Automated Data Analysis Pipeline (ADAP) chromatogram module and peaks were detected via the ADAP wavelets algorithm. Peaks were aligned across all samples via the Random sample consensus aligner module, gap-filled, and assigned identities using an exact mass MS1( ±15ppm) and retention time RT ( ±0.5min) search of our in-house MS1-RT database. Peak boundaries and identifications were then further refined by manual curation. Peaks were quantified by area under the curve integration and exported as CSV files. If stable isotope tracing was used in the experiment, the peak areas were additionally processed via the R package AccuCor 2 to correct for natural isotope abundance. Peak areas for each sample were normalized by the measured area of the internal standard trifluoromethanesulfonate (present in the extraction buffer) and by the number of cells present in the extracted well.

#### *In vitro* GPT activity assay

Tumors were homogenized in 2 mL tubes with stainless steel beads on a TissueLyser LT for 1 min at max speed, followed by a 1 min break, three consecutive times. For every mg of tissue, 10 μL of ALT assay buffer from the Alanine Transaminase Activity Assay Kit (Abcam, ab105134) was used to process the sample. Samples then were clarified by centrifugation at top speed at 4°C, and enzymatic activity assayed following the manufacturer’s instructions. For sample normalization, protein quantification was done using the DC protein assay kit (Biorad, 5000111).

#### siRNA transfection

Non-targeting (NT), mouse *Gpt2*-targeting and human *GPT2*-targeting, and human c-Myc-targeting siRNAs (Dharmacon, D-001810-01-20; L-055921-01-0010, L-004173-01-000, L-003282-02-0005) were dissolved in RNAse-free H2O (Invitrogen, AM9938) at 20 μM and stored at −80°C in single use aliquots.

siRNA knockdown of mouse *Gpt2* and human *GPT2* was done by seeding cells at 100,000 (EC4 and Tong) or 150,000 (HepG2) per 6 well in 2 mL high glucose DMEM +10%FBS + NEAA and transfecting 30 pmol of siRNAs per well with 5.5 μL of lipofectamine RNAi MAX (Invitrogen, AM9938) on the following day, according to the manufacturer’s instructions. 24 h post-transfection cells were lifted, passed through 70 μm cell strainers (Corning, 352350), and seeded for growth assays and knockdown validation (see below).

siRNA knockdown of *MYC* was done by seeding HepG2 cells at 150,000 per 6 well in 2 mL high glucose DMEM +10%FBS + NEAA and transfecting 30 pmol of siRNAs per well with 5.5 μL of lipofectamine RNAi MAX (Invitrogen, AM9938) the following day, according to the manufacturer’s instructions. 48 h post-transfection cells were lifted, passed through 70 μm cell strainers (Corning, 352350), and seeded for growth assays and knockdown validation (see below). Importantly, prior to seeding for transfection, HepG2 cells were maintained at 70–90% confluency to ensure viability and growth post-transfection.

#### Cell proliferation assays

For assays done on MYC-high/low EC4 cells in Figures 1J and 1K, we pretreated cells ±10 ng/mL doxycycline (dissolved in water; Fisher, BP2653-5) in high glucose DMEM +10% FBS + NEAA for 3 days, changing the media every 48 h. For alanine growth assays done on the EC4, SNU475, PLC5, and TONG cell lines in Figures 1J, 1K, and 1M, cells were cultured in DMEM without glucose/glutamine/pyruvate/phenol red (Gibco, A14430-01) supplemented with 10% dialyzed FBS (Gibco, 26400044), 5 mM or 500 μM glucose (Sigma, G8769-100ML), 500 μM glutamine (Sigma, G3126-100G), and the indicated alanine concentrations (A7627-100G). Cells were seeded in clear bottom 96 well plates (Corning, 3603) in 200 μL media at a density of 1000 cells per well, and brightfield images were taken an hour after seeding to measure day 0 cell counts. 100μL of media was removed, and replaced with fresh media 2 days after seeding, and cells were then stained 3 days after seeding with propidium iodide (PI) (Invitrogen, P3566) and Hoechst 33342 (Biotium, 40046) at a final concentration of 1 μg/mL and 100 ng/mL, respectively, and were imaged 4 days after seeding for Hoechst and PI fluorescence on a Biotek high content microscopy plate reader. For these studies, glutamine was dissolved at 200 mM in water and frozen in single use aliquots at −80°C, and media was made fresh for each experiment.

Alanine proliferation assays on HepG2 cells in Figure 1M were seeded at 28,000 cells per well in 6 well plates (VWR, 10062-892). Cells were plated in 2 mL of DMEM without glucose/glutamine/pyruvate/phenol red supplemented with 10% dialyzed FBS, 5 mM or 500 μM glucose, 500 μM glutamine, 0 or 500 μM alanine, and 100μL of media was removed, and replaced with fresh media 2 days after seeding. After 4 days the supernatant containing non-adherent cells, and adherent cells were collected and counted by trypan blue staining (Invitrogen, T10282). For these studies glutamine was dissolved at 200 mM in water and frozen in single use aliquots at −80°C, and media was made fresh for each experiment.

For proliferation assays done on MYC-low EC4 cells in Figure S1C, we pretreated cells with 10 ng/mL doxycycline (Fisher, BP2653-5) in high glucose DMEM +10% FBS + NEAA for 3 days, changing the media every 48 h. EC4 cells were seeded in clear bottom 96 well plates in 200 μL media at a density of 1000 cells per well in DMEM without glucose/glutamine/pyruvate/phenol red (Gibco, A14430-01) supplemented with 10% dialyzed FBS (Gibco, 26400044), 0 or 500 μM alanine (A7627-100G), as well as the indicated glutamine (Sigma, G3126-100G) and glucose (Sigma, G8769-100ML) concentrations. 100μL of media was removed, and replaced with fresh media 2 days after seeding, and cells were then stained 3 days after seeding with PI (Invitrogen, P3566) and Hoechst 33342 (Biotium, 40046) at a final concentration of 1 μg/mL and 100 ng/mL, respectively, and were imaged 4 days after seeding for Hoechst and PI fluorescence on a Biotek high content microscopy plate reader. For these studies, glutamine was dissolved in water at 200 mM, stored at 4°C, and used within 2 weeks to make fresh media for each experiment.

For alanine proliferation assays in Figures 3D, 3E, and S1H, cells were cultured in DMEM without glucose/glutamine/pyruvate/phenol red supplemented with 10% dialyzed FBS, 5mM or 500 μM glucose, 0 or 500 μM alanine and 400 μM glutamine for Tong cells and 350 μM glutamine for HepG2 cells. Tong cells were seeded at 1000 cells per well in clear bottom 96 well plates, and HepG2 cells were seeded at 5000 cells per well in Falcon 96 well plates (Cat# 353072), in 200 μL of the indicated media. 100μL of media was removed and replaced with fresh media 2 days after seeding. Tong cells were stained 3 days after seeding with PI (1 μg/mL, final concentration) and Hoechst (100 ng/mL, final concentration) and imaged 4 days after seeding for Hoechst and PI fluorescence on a Biotek high content microscopy plate reader. HepG2 cell counts were quantified by using the CYQUANT cell proliferation assay (ThermoFisher, C7026) 4 days after seeding. Glutamine was dissolved in water at 200 mM, stored at 4°C, and used within 2 weeks of dissolving and media was made fresh for each experiment.

For siRNA proliferation assays in MYC-high EC4 cells (Figure 3C), cells were collected 1 day post-transfection and seeded at 1000 cells per well in clear bottom 96 well plates in 200 μL DMEM without glucose/glutamine/pyruvate/phenol red supplemented with 10% dialyzed FBS, 500 μM glucose, 500 μM glutamine, 500 μM alanine, and brightfield images were taken an hour after seeding to measure day 0 cell counts. Methyl pyruvate (Thermo scientific, A13966.14) and dimethyl glutamate (Thermo scientific, L03764.06) were dissolved in water at 500 mM and added to the indicated conditions at 500 μM. Media was changed every 2 days by removing 100μL of media and replenishing with the same volume of fresh media, and cells were then stained 3 days after seeding with PI (Invitrogen, P3566) and Hoechst 33342 (Biotium, 40046) at a final concentration of 1 μg/mL and 100 ng/mL, respectively. Cells were then imaged 4 days after seeding for Hoechst and PI fluorescence on a Biotek high content microscopy plate reader. siRNA proliferation assays in Tong cells (Figure 3E) were done identically, except cells were seeded in media containing 400 μM glutamine. siRNA proliferation assays in HepG2 cells (Figure 3D) were done identically, except cells were cultured in media containing 350 μM glutamine and seeded in Falcon 96 well plates at 2500 cells per well. HepG2 cells were quantified by using the CYQUANT cell proliferation assay 4 days after seeding. siRNA studies done in MYC-low EC4 cells (Figure S3A) were done identically to those in Figure 3B, except we pretreated cells with 10 ng/mL doxycycline (Fisher, BP2653-5) in high glucose DMEM +10% FBS + NEAA for 3 days, changing the media every 48 h by removing 100μL of media and replenishing with the same volume of fresh media. For siRNA studies, glutamine was dissolved in water at 200 mM, stored at 4°C, and used within 2 weeks to make fresh media for each experiment.

For siRNA knockdown validation, cells were seeded in high glucose DMEM +10% FBS + NEAA at 50,000 cells per well in 6 well plates (EC4 and Tong) or 100,000 cells per well in 6 well plates (HepG2), and RNA or protein was collected 2 days after seeding.

For L-cycloserine dose response assays (Figure 4A), EC4 cells were pretreated ±10 ng/mL doxycycline in high glucose DMEM +10% FBS + NEAA for 3 days, replenishing media every 2 days. For the proliferation assay, cells were seeded at 1000 cells per well in a clear bottom 96 well plate in 200 μL DMEM no glucose/glutamine/pyruvate/phenol supplemented with 10% dialyzed FBS, 500 μM glucose, 500 μM glutamine, and 500 μM alanine at the indicated L-cycloserine levels. Brightfield images were taken an hour after seeding to measure day 0 cell counts. 100μL of media was removed and replaced with fresh media 2 days after seeding, and cells stained 3 days after seeding with PI and Hoechst at a final concentration of 1 μg/mL and 100 ng/mL, respectively. Cells were imaged on a Biotek high content microscopy plate reader for PI and Hoechst fluorescence 4 days after seeding. For L-cycloserine response assays, glutamine was dissolved at 200 mM in water and frozen in single use aliquots at −80°C and media was made fresh for each experiment.

To quantify total cells and dead cells in EC4, PLC5, SNU475, and Tong experiments, Hoechst 33342 and PI puncta were counted using the BioTek Gen5 Software, and cell counts were defined as day 4 (# Hoechst puncta – # PI puncta). We quantified normalized cell counts as day 4 (# Hoechst puncta – # PI puncta)/day 0 brightfield counts. Cell death was quantified as (# PI puncta/# Hoechst puncta) x 100%. HepG2 cell counts in Figures S1D, S1H, and 3D were quantified as day 4 CYQUANT cells counts/day 0 brightfield counts.

#### High content microscopy

Cell growth assays were done on the Biotek Cytation 5 high content microscope (Agilent). The machine uses a Blackfly camera (BFLY-U3–2356M) equipped with a Sony IMX249 sensor. An Olympus 4X objective (UPLFLN4×) was used in conjunction with a Laser auto-focus filter cube (Biotek, 1225010), RFP filter cube (Biotek, 1225103) and a DAPI filter cube (Biotek, 1225100) to capture brightfield, PI, and Hoechst images.

#### RNA extraction and cDNA synthesis

RNA was extracted using the QIAshredder Kit (Qiagen, 79656) and the Rneasy Mini Kit (Qiagen, 74106) according to the manufacturer’s instructions. cDNA was then synthesized using the High Capacity RNA-to-cDNA Kit (Applied Biosystems, 4387406).

#### Quantitative PCR (qPCR)

qPCR reactions were set up using TaqMan Fast Advanced Master Mix (Applied Biosystems, 4444557) in optical 384 well plates (Applied Biosystems, 4309849). The following Taqman probes from Thermo fisher were used: Mm00558028_m1 Gpt2, Mm02619580_g1 Actb, Hs99999903_m1 ACTB, Hs00370287_m1 GPT2. Relative transcript levels were calculated using the ΔΔCt method, normalizing to the reference gene Actb.

#### Immunoblotting

Proteins were extracted in RIPA buffer (50mM Tris-pH7.4, 150nM NaCl. 0.5% Triton, 0.2% Sodium deoxycholate) supplemented with protease and phosphatase inhibitors (Roche, 11697498001, 4906845001). Lysates were homogenized by passing them through 28.5 G insulin syringes (BD, 329424) 5–10 times and clarified by centrifuging at 17,000 x g for 15 min at 4°C. Supernatants were collected and transferred to new tubes, and protein content quantified using the DC assay (BioRad, 5000111). Samples were then prepared in 4X SDS sample buffer containing 100mM DTT (Fisher, BP172-25G).

Samples were run on 4–12% SDS-PAGE gels (Invitrogen, NW04122BOX) and transferred to nitrocellulose membranes (Invitrogen, IB23002). Membranes were blocked with 5% milk dissolved in Tris-Buffered Saline/0.1% Tween 20 (TBST) for 1 h at room temperature and then incubated with primary antibodies dissolved in 5% milk/TBST overnight at 4° on a shaker. The following day membranes were washed 3 times in TBST for 5 min, and then incubated with HRP-conjugated secondary antibody diluted 1:5000 in 5% milk/TBST for 2 h. After incubation, membranes were washed 3 times in TBST, and signal visualized using ECL (Bio-rad, 170–5061) or ECL Prime (Amersham, RPN2232).

The antibodies used and their dilutions in 5% milk/TBST follow: α-GPT1 (Abcam, ab202083, 1:1000), α-GPT2 (Sigma, HPA051514, 1:1000), α-β-actin-HRP (Santa Cruz, sc-47778 HRP, 1:5000), α-*c*-MYC (Abcam, ab32072, 1:1000), α-Vinculin (Cell Signaling Technologies, 13901, 1:1000), α-ATF4 (Cell Signaling Technologies, 11815, 1:1000), α-eIF2α (Cell Signaling Technologies, 9722, 1:1000), α-P-eIF2α (Cell Signaling Technologies, 3597, 1:1000), α-Rabbit IgG-HRP (Promega, W4011, 1:5000).

### QUANTIFICATION AND STATISTICAL ANALYSIS

Statistical analyses were done using the Prism software (GraphPad), and details on the tests used and the sample size (n) and what n represents can be found in the figure legends. All cell proliferation assays show the mean ± SEM of cell counts after 4 days of growth, divided by the initial seeding density. Cell counts for experimental conditions are shown relative to control condition, which was normalized to 1 for each experiment. Multiple one sample *t* tests with a Bonferroni correction were performed to compare normalized cell counts to the control value of 1. Cell death levels (Figures 1J and 1K) are shown as the mean ± SEM of the percentage PI positivity, and a one-way ANOVA with Dunnet’s multiple-comparison test was performed to compare experimental conditions to the 0 alanine control. Metabolomics data from the LT2-MYC model (Figure 1D) shows normalized mean metabolite abundance ± SEM, and a two-sample *t* test was performed to compare metabolite abundance between the 2 groups. Alanine abundance from matched HCC tumors and non-tumor tissue in Figure 1F was LOG_2_ transformed, and a matched pairs *t* test was performed to compare relative alanine abundance within each subject. Human serum alanine abundance in Figure 1G is shown as mean ± SEM, and a two-sample *t* test was performed to compare relative alanine abundance between the two groups. Transcript abundance of HCC tumor samples in Figure 1H was LOG2 transformed, and the correlation coefficient and R^2^ between GPT2 and MYC transcript levels was calculated. Labeling efficiency in the alanine tracing studies (Figures 2 and S2) denotes the percentage of the total metabolite pool that had at least one heavy isotope label incorporated in matched tumor and non-tumor tissue from the same animal. A matched pairs *t* test was performed to compare metabolite labeling efficiency within each animal. Percentage survival was plotted in Figures 3H, 4E, and 4I, and significance was determined with a log-rank test. L-Cycloserine response assay in Figure 4A for the treated conditions show mean normalized cell counts ± SEM; a two-sample *t* test with a Holm-Sidak multiple-comparisons correction was performed to determine significance. The mean ± SEM of the ratio of GPT2 activity to total protein content is shown in Figure 4C, and a two-sample *t* test was performed to determine significance. The mean ± SEM of liver weights for each condition is shown in Figure 4D, and a two-sample *t* test was performed to determine significance. The mean ± SEM of the tumor volume of each mouse still enrolled in the study is shown in Figure 4H, and a two-sample *t* test with a Holm-Sidak multiple-comparisons correction was performed to determine significance. All qPCR data shows the mean ± SEM of transcript abundance calculated using the ΔΔCt method with the control condition set to 1; a one sample *t* test was performed to compare the experiment condition to the control value of 1. Western blot quantification of MYC knockdown efficiency show the mean ± SEM of the ratio of c-MYC/β-actin signal, with the siNT condition set to 1 for each experiment; a one sample test comparing the ratio of c-MYC to β-actin in the siMYC condition to the control value of 1 was then performed. Significance was determined based on if a P-value was less than 0.05. There was no method used to determine whether the data met the assumptions of the statistical approach, and there was no strategy for stratification, randomization, or sample size estimation.

## Supplementary Material

1

Supplemental information can be found online at https://doi.org/10.1016/j.celrep.2026.117107.

## Figures and Tables

**Table:** KEY RESOURCES TABLE

REAGENT or RESOURCE	SOURCE	IDENTIFIER
Antibodies		
α-GPT2	Sigma	Cat# HPA051514; RRID: AB_2681516
α-GPT1	Abcam	Cat# ab202083; RRID: AB_2915976
α-*c*-MYC, clone Y69	Abcam	Cat# ab32072; RRID: AB_731658
α-β-actin-HRP	Santa Cruz	Cat# sc-47778; RRID: AB_2714189
α-Vinculin	Cell Signaling Technologies	Cat# 13901; RRID: AB_2728768
α-ATF4	Cell Signaling Technologies	Cat# 11815; RRID: AB_2616025
α-eIF2α	Cell Signaling Technologies	Cat# 9722; RRID: AB_2230924
α-P-eIF2α	Cell Signaling Technologies	Cat# 3597; RRID: AB_390740
α-Rabbit IgG-HRP	Promega	Cat# W4011; RRID: AB_430833

Bacterial and virus strains		

NEB^®^ Stable Competent *E. coli* (High Efficiency)	New England Biolabs	Cat# C3040H

Chemicals, peptides, and recombinant proteins		
Doxycycline	Fisher Scientific	Cat# BP2653-5
L-Cycloserine	MedChemExpress	Cat# HY-B1122
Trypan blue	Invitrogen	Cat# T10282
DTT	Fisher Scientific	Cat# BP172-25G
Heparin	McKesson	Cat# NDC 63739-931-14
Lipofectamine RNAi MAX	Invitrogen	Cat# AM9938
^13^C_3_;^15^N-alanine	Cambridge Isotopes	Cat# CNLM-534-H-PK
Trifluoromethanesulfonate	Sigma	Cat# 422843-5G
Water; LC-MS grade	Fisher Scientific	Cat# W6-212
Methanol; LC-MS grade	Fisher Scientific	Cat# A456-500
Acetonitrile; LC-MS grade	Fisher Scientific	Cat# A955-4
Non-essential amino acids	UCSF Cell Culture Facility	Cat# CCFGA001-22BU01
L-Alanine	Sigma	Cat# A7627-100G
L-Glutamine	Sigma	Cat# G3126-100G
D-(+)-Glucose solution	Sigma	Cat# G8769-100ML
Methyl-pruvate	Thermo scientific	Cat# A13966.14
Dimethyl-glutamate	Thermo scientific	Cat# L03764.06
Hoechst 33342	Biotium	Cat# 40046
Propidium Iodide	Invitrogen	Cat# P3566

Critical commercial assays		
Alanine Transaminase Activity Assay Kit	Abcam	Cat# ab105134
DC protein assay kit	Biorad	Cat# 5000111
QIAshredder Kit	Qiagen	Cat# 79656
CYQUANT cell proliferation assay	ThermoFisher	Cat# C7026
Rneasy Mini Kit	Qiagen	Cat# 74106
High Capacity RNA-to-cDNA Kit	Applied Biosystems	Cat# 4387406
TaqMan Fast Advanced Master Mix	Applied Biosystems	Cat# 4444557
Mouse Gpt2 taqman probe	Thermo fisher	Cat# Mm00558028_m1 Gpt2
Mouse Actb taqman probe	Thermo fisher	Cat# Mm02619580_g1 Actb
Mouse Gpt2 taqman probe	Thermo fisher	Cat# Hs99999903_m1 ACTB
Mouse Actb taqman probe	Thermo fisher	Cat# Hs00370287_m1 GPT2

Deposited data		
The Cancer Genome Atlas (HCC)	Ally et al.^5^	https://gdc.cancer.gov/; https://www.cbioportal.org/study/summary?id=lihc_tcga
RNA sequencing and ChIP sequencing in the LT2-MYC model	Kress et al.^18^	GEO: GSE76078
LT2-MYC metabolomics data	Anderton et al.^15^	https://pmc.ncbi.nlm.nih.gov/articles/PMC5376764/#embr201643068-sup-0003
^13^C_3_;^15^N-alanine tracing in the LT2-MYC model	https://doi.org/10.21228/M8TK1K	NMDR: ST004169

Experimental models: Cell lines		
EC4	Cao et al.^50^	N/A
HepG2	ATCC	HB-8065; RRID: CVCL_0027
Tong	Xin Chen (University of Hawai’i Cancer Center)	RRID: CVCL_V640
PLC5	Xin Chen (University of Hawai’i Cancer Center)	CRL-8024; RRID: CVCL_0485
SNU475	Xin Chen (University of Hawai’i Cancer Center)	CRL-2236; RRID: CVCL_0497

Experimental models: Organisms/strains		
C57BL/6 mice	JAX	Cat# 000664
Nude mice	JAX	Cat# 002019
LT2-MYC mice	Shachaf et al.^51^	N/A
GPT2 FLOX mice	Martino et al.^10^	N/A

Recombinant DNA		
pT3-MYC-IRES-CRE	This study.	N/A
px330-p53	Addgene	Cat# 92046
pSB100X	Addgene	Cat# 34879

Software and algorithms		
Image Lab	Bio-rad	https://www.bio-rad.com/en-us/product/image-lab-software?ID=KRE6P5E8Z
Prism	GraphPad	https://www.graphpad.com/
msconvert	ProteoWizard	https://proteowizard.sourceforge.io/download.html
MZmine 2 software package	Github	http://mzmine.github.io/
R package AccuCor 2	Github	https://github.com/lparsons/accucor
BioTek Gen5 Software for Imaging & Microscopy	Agilent	https://www.agilent.com/en/product/cell-analysis/cell-imaging-microscopy/cell-imaging-microscopy-software/biotek-gen5-software-for-imaging-microscopy-1623226?srsltid=AfmBOorqyrNTWIselZZ5dCAZIUrMqIsTA5pCTEkRPb9ukj-fGgoqPlhf

Other		
ECL	Bio-rad	Cat# 170-5061
ECL Prime	Amersham	Cat# RPN2232
Protease inhibitors	Roche	Cat# 11697498001
Phosphatase inhibitors	Roche	Cat# 4906845001
28.5 G insulin syringes	BD	Cat# 329424
4–12% SDS-PAGE gels	Invitrogen	Cat# NW04122BOX
Nitrocellulose membranes	Invitrogen	Cat# IB23002
Pump 11 Elite Dual Syringe Infusion Pump	Harvard apparatus	Cat# 70-4501
Catheters	Instech	Cat# C20PU-MJV2014
Sutures	Surgical Specialties	Cat# SP117
Savant DNA120 SpeedVac System	ThermoFisher	Cat# DNA120-115
5 mm stainless-steel beads	Qiagen	Cat# 69989
TissueLyser LT	Qiagen	Cat# 85600
Vanquish UHPLC	Thermo Scientific	N/A
SeQuant ZIC-pHILIC Polymeric column (2.1 3 150 mm 5 mm)	EMD Millipore	Cat#: 150460
Q-Exactive mass analyzer	Thermo Scientific	N/A
DMEM	Gibco	Cat# 11995-065
DMEM no glucose/glutamine/pyruvate/phenol red	Gibco	Cat# A14430-01
FBS	Gibco	Cat# 10437-028
Dialyzed FBS	Gibco	Cat# 26400044
70 μm cell strainers	Corning	Cat# 352350
Clear bottom 96 well plates	Corning	Cat# 3603
6 well plates	VWR	Cat# 10062-892
Falcon 96 well plates	Corning	Cat# 353072
Optical 384 well plates	Applied Biosystems	Cat# 4309849
Biotek Cytation 5 high content microscope	Agilent	N/A
Blackfly camera for biotek	Blackfly	Cat# BFLY-U3-2356M
Sony IMX249 sensor	Sony	Cat# IMX249LLJ-C
Olympus 4X objective	Olympus	Cat# UPLFLN4X
Laser auto-focus filter cube	Biotek	Cat# 1225010
RFP filter cube	Biotek	Cat# 1225103
DAPI filter cube	Biotek	Cat# 1225100

## Data Availability

Metabolomics data were uploaded to the National Metabolomics Data Repository^49^ under NMDR: ST004169. This paper does not report original code. Any additional information required to reanalyze the data reported in this paper is available from the lead contact upon request.

## Highlights

- MYC liver tumors catabolize alanine to support bioenergetics, biosynthesis, and gluconeogenesis
- Genetic ablation of GPT2 is sufficient to slow MYC-driven liver tumorigenesis
- L-Cycloserine, a compound that inhibits GPT2, slows growth of established liver tumors
