# Supplementary material for: Alanine catabolism as a targetable vulnerability for MYC-driven liver cancer
Source: Cell Rep. Author manuscript; Available in PMC 2026 Jun 13. (PMC13264379; doi:10.1016/j.celrep.2026.117107)
Supplement: 1 [file NIHMS2170755-supplement-1.pdf]

Cell Reports, Volume *45*

Supplemental information

Alanine catabolism as a targetable vulnerability for MYC-driven liver cancer

Tonatiuh Montoya, Joyce V. Lee, Longhui Qiu, Abigail Krall, Nedas Matulionis, Yurim Seo, Brian N. Finck, Robin K. Kelley, Heather Christofk, and Andrei Goga

Supplementary Figure 1

A

genes	Myc_indep_UP	Myc_dep_UP	Myc_indep_DN	Myc_dep_DN
Odc1	no	yes	no	no
Gpt	no	no	yes	no
Gpt2	no	no	no	no

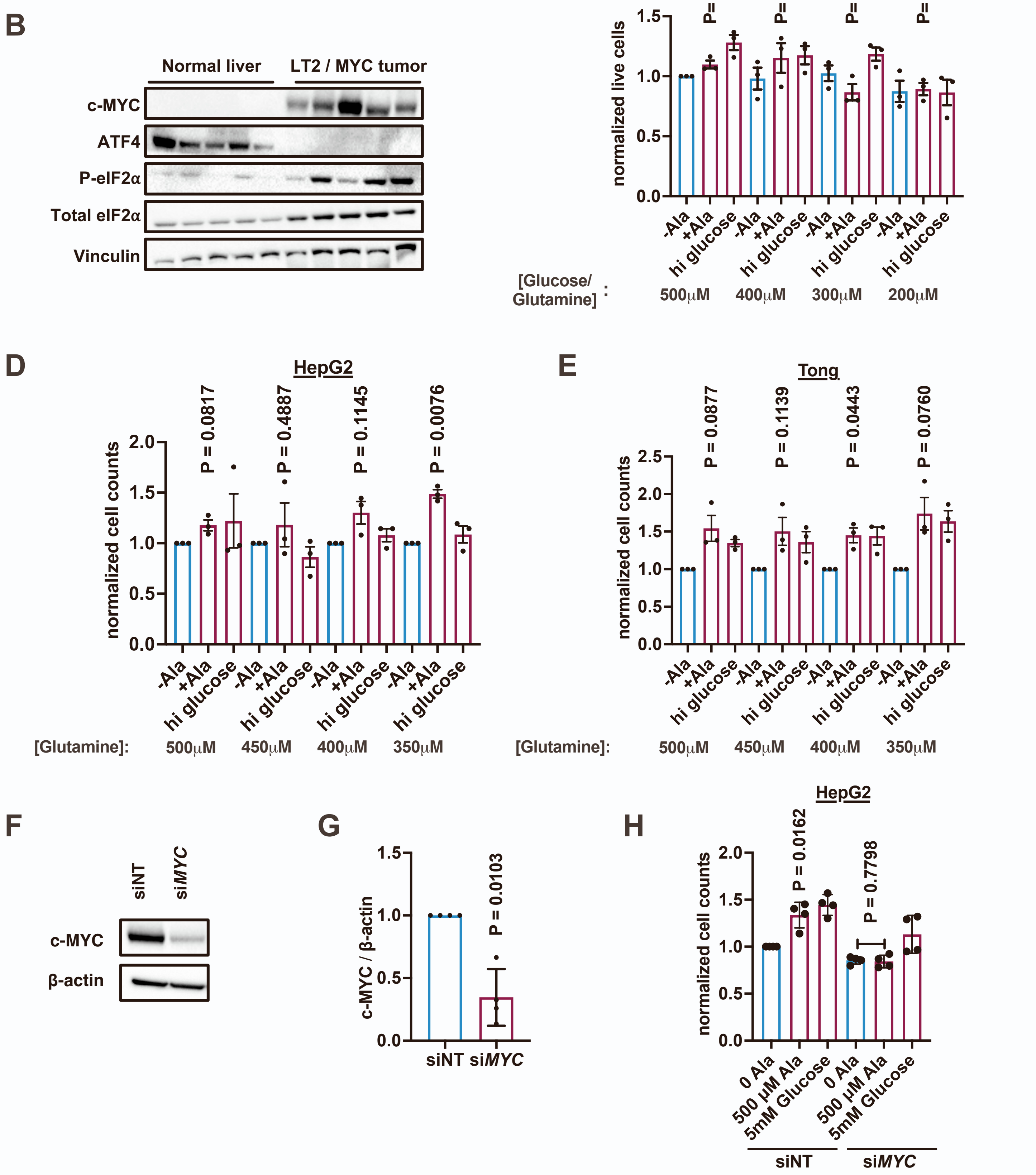

Supplementary Figure 1. Expression profiling of LT2-MYC tumors, glutamine dependency of alanine catabolism, and MYC-dependency of alanine catabolism in HepG2 cells (related to Figure 1) (A) Summary of ChIP-Seq and RNA-seq study performed in the LT2-MYC tumor model, adapted from Kress et al. [S1] A “yes” or “no” denotes whether a transcript is regulated in a MYC-dependent (“Myc_dep”) or MYC-independent manner (“Myc_indep”). “DN” denotes downregulation, and “UP” denotes upregulation. (B) Immunoblotting of LT2-MYC tumors and control liver for unfolded protein response pathway proteins. (C) Alanine proliferation response of MYC low EC4 cells in the indicated glucose and glutamine concentrations (n=3 independent experiments per condition). (D-E) Alanine proliferation response of HepG2 (D) and Tong (E) cells across a range of glutamine concentrations (n=3 independent experiments per condition). (F) Representative western blot confirmation of MYC knockdown in HepG2 cells. Quantification of MYC knockdown efficiency in (G) and quantification of HepG2 cell counts after 4 days of growth in 350 µM glutamine 500 µM alanine media +/- MYC knockdown in (H). n=4 independent experiments for (G-H). Plots in (C-E) and (H) show mean +/- SEM with the 0 alanine condition for each glutamine condition set to 1. Significance in (C-E) calculated using multiple one-sample t-tests comparing each condition to a control value of 1. n=3 independent experiments for each condition. For (G), the expression of c-MYC was divided by that of β-actin and set to 1 for the siNT condition; a one sample test comparing the ratio of c-MYC to β-actin in the siMYC condition to a control value of 1 was then performed. For (H), the relative cell counts of the 0 alanine condition were set to 1 and a one sample t-test was used to compare cell counts of siNT cells in 500 µM Alanine to a control value which was set to 1; a two-sample t-test was then used to compare cell counts of siMYC cells in the 0 alanine media to the 500 µM condition.

Supplementary Figure 2

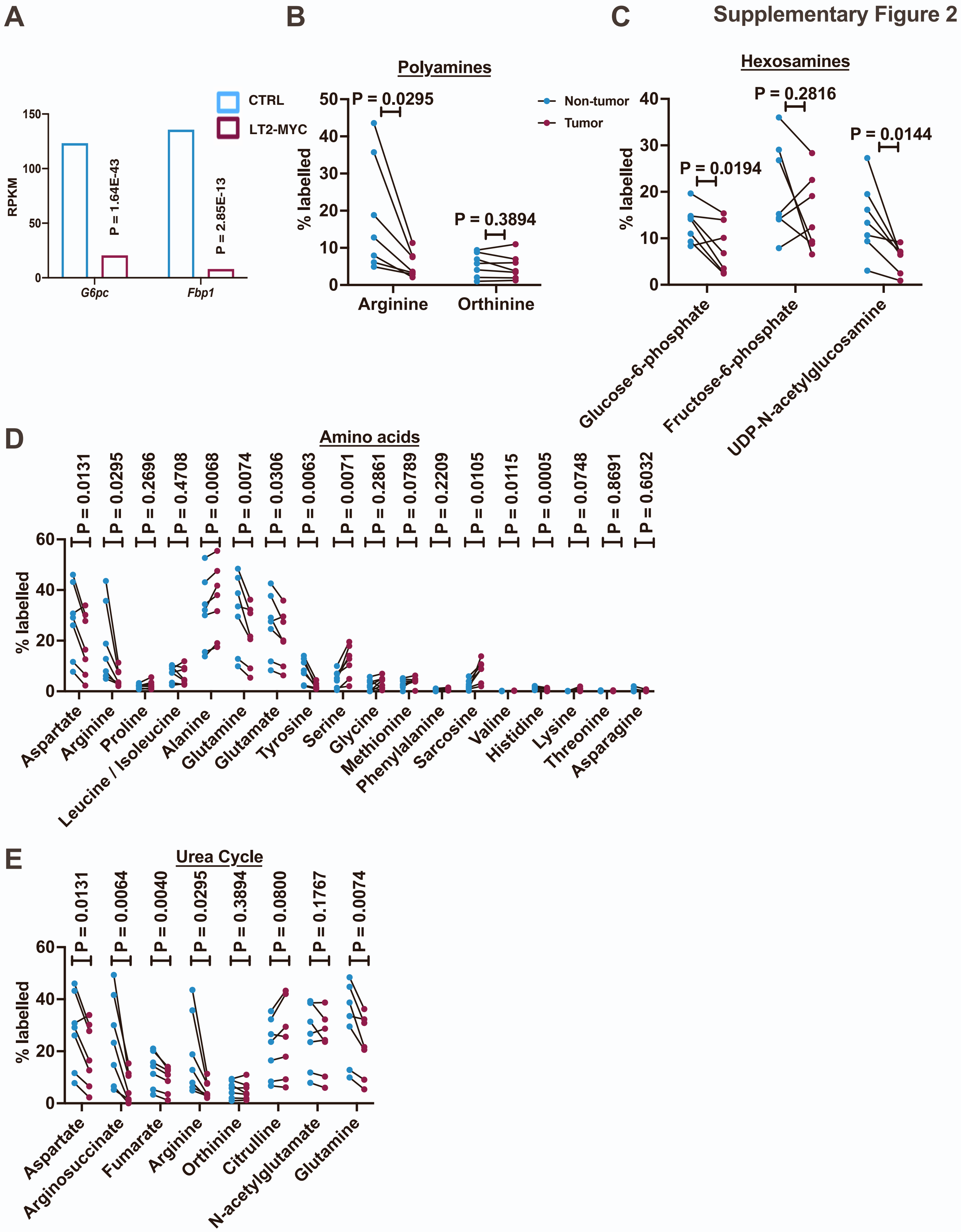

Supplementary Figure 2. Alanine is a substrate for *in vivo* tumor metabolism (related to Figure 2) (A) Transcript levels of *Fbp1* and *G6pc1* by RNA-seq, from reference [S1]; n=11-16 per condition. Shown is the fractional contribution of $^{13}C_3$; ^{15}N-alanine to polyamines (B), hexosamines (C), amino acids (D), and the urea cycle (E). n=7 mice for each group. Plot in A shows exonic RPKM values from RNA-seq. Plots for (B-E) show metabolite abundance in matched tumor and non-tumor liver tissue for each animal. Significance for (E) calculated in reference [S1], which used DESeq2 to calculate adjusted P-values for RNA-seq. Significance for (B-E) was determined using a matched pairs t-test.

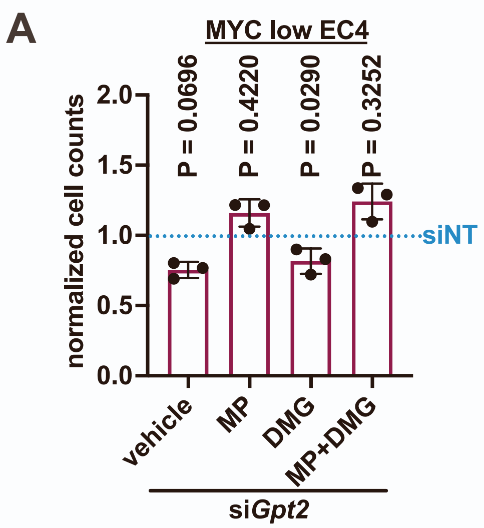
A
MYC low EC4
normalized cell counts
2.0
1.5
1.0
0.5
0.0
P = 0.0696
P = 0.4220
P = 0.0290
P = 0.3252
siNT
vehicle
MP
DMG
MP+DMG
siGpt2

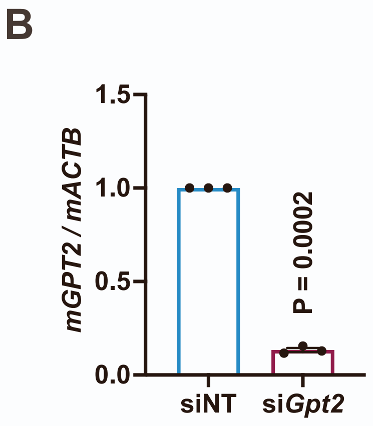
B
mGPT2 / mACTB
1.5
1.0
0.5
0.0
P = 0.0002
siNT
siGpt2

Supplementary Figure 3. MYC-dependency of *Gpt2*-dependent alanine catabolism in EC4 cells (related to Figure 3) (A) Proliferation response of MYC low EC4 cells +/- *Gpt2* knockdown in 500 µM alanine / glutamine media upon treatment with 500 µM methyl-pyruvate and / or dimethyl-glutamate (n=3 independent experiments each group). The blue dotted line represents the proliferation of control siNT cells which was normalized to 1. (C) Quantification of *Gpt2* knockdown efficiency in MYC low EC4 cells by qPCR (cDNA from n=3 independent experiments). Plots in (A-B) show mean +/- SEM of normalized cell counts or normalized transcript levels where indicated. Significance in (A) determined multiple one-sample t-tests with a Bonferroni correction and the non-targeting control set to 1; a one-sample t-test with the non-targeting control set to 1 in (B).

Supplementary Figure 4

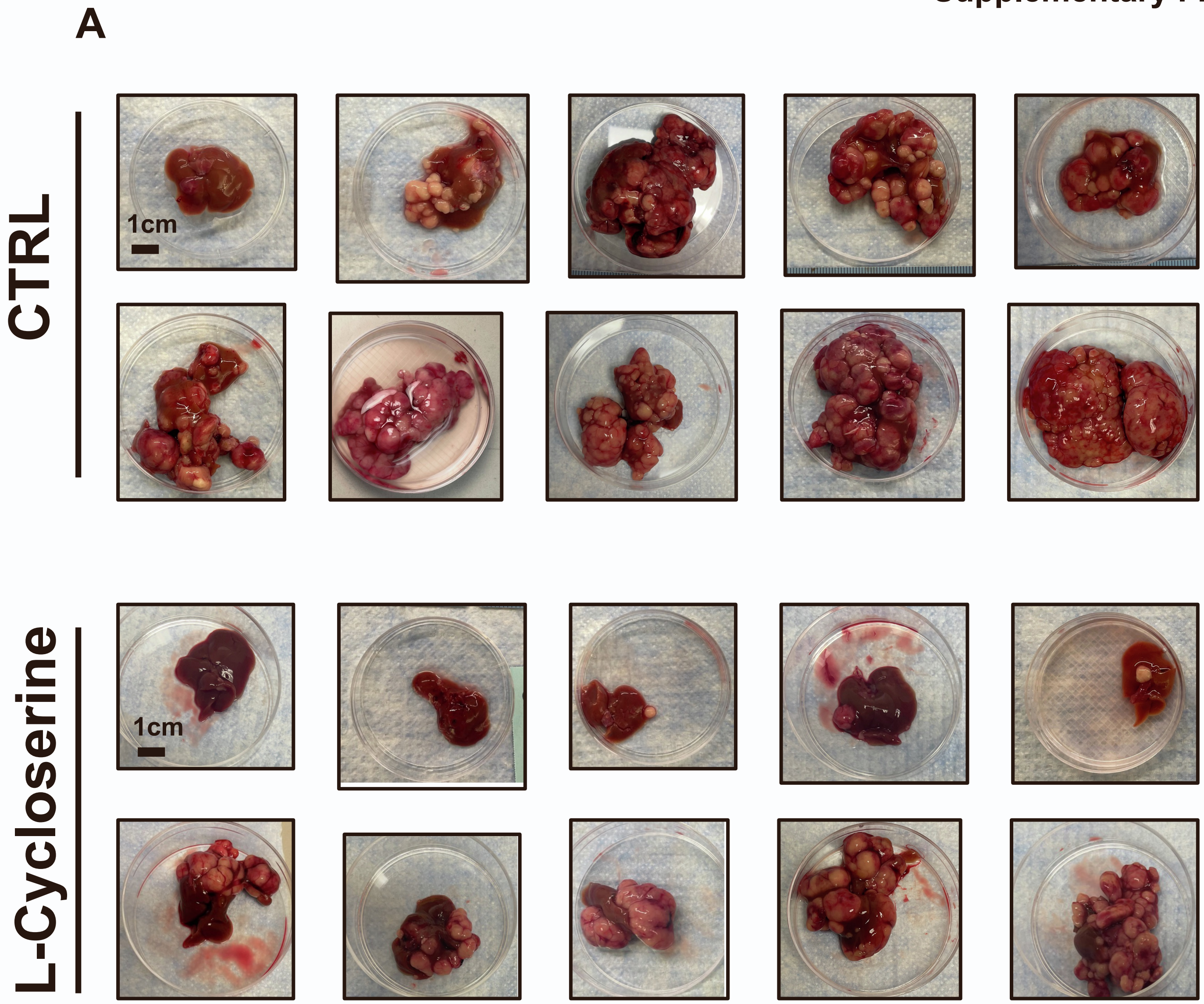

Supplementary Figure 4. Images of L-Cycloserine-treated LT2-MYC livers (related to Figure 4)
(A) Representative liver tumor images from the LT2-MYC L-Cycloserine study in Figure 4E. Livers were collected when mice reached ethical endpoint or at the end of the study, which was 12 weeks in length. Shown are 10 representative livers for each condition. Scale bar, 1cm.

Supplementary References

S1. Kress, T.R., Pellanda, P., Pellegrinet, L., Bianchi, V., Nicoli, P., Doni, M., Recordati, C., Bianchi, S., Rotta, L., Capra, T., et al. (2016). Identification of MYC-Dependent Transcriptional Programs in Oncogene-Addicted Liver Tumors. Cancer Res. *76*, 3463–3472. https://doi.org/10.1158/0008-5472.CAN-16-0316.
